# Targeting microglial autophagic degradation of the NLRP3 inflammasome for identification of thonningianin A in Alzheimer’s disease

**DOI:** 10.1186/s41232-022-00209-7

**Published:** 2022-08-03

**Authors:** Xiao-Gang Zhou, Wen-Qiao Qiu, Lu Yu, Rong Pan, Jin-Feng Teng, Zhi-Pei Sang, Betty Yuen-Kwan Law, Ya Zhao, Li Zhang, Lu Yan, Yong Tang, Xiao-Lei Sun, Vincent Kam Wai Wong, Chong-Lin Yu, Jian-Ming Wu, Da-Lian Qin, An-Guo Wu

**Affiliations:** 1 Sichuan Key Medical Laboratory of New Drug Discovery and Drugability Evaluation, Luzhou Key Laboratory of Activity Screening and Druggability Evaluation for Chinese Materia Medica, Key Laboratory of Medical Electrophysiology of Ministry of Education, School of Pharmacy, Southwest Medical University, Luzhou, 646000 China; 2 Department of Neurosurgery Sichuan Provincial People’s Hospital, University of Electronic Science and Technology of China, Chengdu, 610000 China; 3 School of Pharmaceutical Sciences, Hainan University, Haikou, 570228 China; 4 State Key Laboratory of Quality Research in Chinese Medicine, Macau University of Science and Technology, Macau, 999078 China; 5 Vascular Surgery Department, Affiliated Hospital of Southwest Medical University, Luzhou, 646000 China

**Keywords:** Thonningianin A, NLRP3 inflammasome, Alzheimer’s disease, Autophagy, AMPK/ULK1, Raf/MEK/ERK

## Abstract

**Background:**

NLRP3 inflammasome-mediated neuroinflammation plays a critical role in the pathogenesis and development of Alzheimer’s disease (AD). Microglial autophagic degradation not only decreases the deposits of extracellular Aβ fibrils but also inhibits the activation of NRLP3 inflammasome. Here, we aimed to identify the potent autophagy enhancers from *Penthorum chinense* Pursh (PCP) that alleviate the pathology of AD via inhibiting the NLRP3 inflammasome.

**Methods:**

At first, autophagic activity-guided isolation was performed to identify the autophagy enhancers in PCP. Secondly, the autophagy effect was monitored by detecting LC3 protein expression using Western blotting and the average number of GFP-LC3 puncta per microglial cell using confocal microscopy. Then, the activation of NLRP3 inflammasome was measured by detecting the protein expression and transfected fluorescence intensity of NLRP3, ASC, and caspase-1, as well as the secretion of proinflammatory cytokines. Finally, the behavioral performance was evaluated by measuring the paralysis in *C. elegans,* and the cognitive function was tested by Morris water maze (MWM) in APP/PS1 mice.

**Results:**

Four ellagitannin flavonoids, including pinocembrin-7-O-[4″,6″-hexahydroxydiphenoyl]-glucoside (PHG), pinocembrin-7-O-[3″-O-galloyl-4″,6″-hexahydroxydiphenoyl]-glucoside (PGHG), thonningianin A (TA), and thonningianin B (TB), were identified to be autophagy enhancers in PCP. Among these, TA exhibited the strongest autophagy induction effect, and the mechanistic study demonstrated that TA activated autophagy via the AMPK/ULK1 and Raf/MEK/ERK signaling pathways. In addition, TA effectively promoted the autophagic degradation of NLRP3 inflammasome in Aβ(1–42)-induced microglial cells and ameliorated neuronal damage via autophagy induction. In vivo, TA activated autophagy and improved behavioral symptoms in *C. elegans*. Furthermore, TA might penetrate the blood-brain barrier and could improve cognitive function and ameliorate the Aβ pathology and the NLRP3 inflammasome-mediated neuroinflammation via the AMPK/ULK1 and Raf/MEK/ERK signaling pathways in APP/PS1 mice.

**Conclusion:**

We identified TA as a potent microglial autophagy enhancer in PCP that promotes the autophagic degradation of the NLRP3 inflammasome to alleviate the pathology of AD via the AMPK/ULK1 and Raf/MEK/ERK signaling pathways, which provides novel insights for TA in the treatment of AD.

**Supplementary Information:**

The online version contains supplementary material available at 10.1186/s41232-022-00209-7.

## Background

Alzheimer’s disease (AD), the most common neurodegenerative disease, accounts for two-thirds of all cases of dementia. It is characterized by the progressive loss of neurons and synapses, as well as the decline of cognitive and memory [10, 23, 59]. The extracellular deposition of β-amyloid (Aβ) in senile plaques and intracellular hyperphosphorylated Tau in neurofibrillary tangles (NFT) are the two main pathological hallmarks of AD [9, 30]. Microglia are a type of resident macrophages located throughout the brain and spinal cord, and they constantly scavenge the central nervous system for plaques and damaged or unnecessary neurons and synapses [37].

Although the inflammatory response is generally beneficial and protects the brain against damage from infectious agents, sustained inflammation is potentially self-damaging [12, 27]. Emerging evidence indicates that aggregated misfolded proteins, such as Aβ and Tau, induce proinflammatory responses and ultimately contribute to the decline of cognition and memory [1, 48]. In addition, the nucleotide-binding oligomerization domain-, leucine-rich repeat- and pyrin domain-containing 3 (NLRP3) inflammasome, the most studied inflammasome, is found to be activated in the brains of AD patients, which is accompanied by the increased release of pro-inflammatory cytokines from microglia [18, 19]. Finally, the sustained proinflammatory responses in microglia further aggravate the pathology of AD and accelerate the progression of AD [5, 19]. Therefore, suppression of the overactivation of NLRP3 inflammasome-mediated neuroinflammation has become a promising therapeutic strategy for AD [57, 58].

Autophagy is a catabolic process of cells that sequesters the cytoplasm, including damaged organelles, unfolding and toxic proteins, and other unnecessary materials, into double membraned vesicles. The formed autophagosome is then fused with a lysosome, and the engulfed components are degraded into amino acids to maintain cell survival [15, 63]. Emerging evidence indicates that deficient autophagy aggravates the pathogenesis of AD [47]. Targeting microglial autophagy for the clearance of misfolded proteins and the degradation of NLRP3 inflammasome is recognized as a promising therapeutic strategy for AD [57]. For example, AICAR, an AMPK activator, was reported not only to degrade extracellular Aβ fibrils but also to suppress organelle stress, such as Aβ-induced activation of the NLRP3 inflammasome and the release of proinflammatory cytokines via autophagy induction [6]. Therefore, the discovery of potent autophagy inducers targeting the degradation of Aβ-induced NLRP3 inflammasome has become a crucial work in the current research.

Traditional Chinese medicine (TCM) has more than 2000 years of history in preventing and treating dementia in China. *Penthorum chinense* Pursh (PCP), Ganhuangcao in Chinese, is a folk medical herb that is mainly distributed in Gulin County, Luzhou City, Sichuan Province. It is commonly used as a kind of health tea in the local region [4, 51]. Emerging modern pharmacological studies show that PCP extract and its derived components exert potent anti-oxidation, anti-tumor, liver protection, and other effects [3, 4, 20, 22, 60, 65, 66, 70]. The chemical analysis of PCP demonstrated that the main components are polyphenols, including rutin, quercetin, and ellagitannin flavonoids [17, 21, 49]. In our previous study, four ellagitannin flavonoids, including pinocembrin-7-O-[4″,6″-hexahydroxydiphenoyl]-glucoside (PHG), pinocembrin-7-O-[3″-O-galloyl-4″,6″-hexahydroxydiphenoyl]-glucoside (PGHG), thonningianin A (TA), and thonningianin B (TB), were identified from PCP to induce autophagy in HUVECs. In the current study, we further validated the autophagy effects of PHG, PGHG, TA, and TB in microglial cells and clarified that TA activated autophagy mainly via the AMPK/ULK1 and Raf/MEK/ERK signaling pathways. Most importantly, we found for the first time that TA promoted the autophagic degradation of the NLRP3 inflammasome in Aβ(1–42)-induced microglia cells and ameliorated the damage of neuronal cells. In addition, TA induced autophagy and improved behavioral functions in *Caenorhabditis elegans* (*C. elegans*). Meanwhile, TA also improved cognitive function and ameliorated the Aβ pathology and inhibited the NLRP3 inflammasome in amyloid precursor protein and mutant human presenilin 1 (APP/PS1) mice. Taken together, the present study provides novel insights for ellagitannin flavonoids, potent autophagy inducers identified from PCP, as new candidates to treat AD in the future.

## Materials and methods

### Chemicals, plasmids, and antibodies

PCP parts, including leaf, stem, and flowers, were purchased from Zi Ning Zhong Yao Ying Pian Co., Ltd. (Chuan20110407, Sichuan, China). The purity of the compounds including PHG, PHGH, TA, and TB determined by Agilent 6230 ultra-high-performance liquid chromatography with diode array detection and time-of-flight tandem mass spectrometry (UHPLC-DAD-TOF/MS) was almost 98% (Additional file 1: Fig. S4A). Aβ(1–42) was obtained from Chinapeptides Co., Ltd. (Shanghai, China). Advanced RPMI 1640 medium, fetal bovine serum (FBS), horse serum (HS), trypsin-EDTA solution, and penicillin-streptomycin (PS) solution (× 100) were purchased from Gibico (Santa Clara, CA, USA). The pEGFP-LC3 and mRFP-GFP tandem fluorescent-tagged LC3 (tfLC3) plasmids were gifted from Prof. Tamotsu Yoshimori (Osaka University, Osaka, Japan). pEGFP-N1-NLRP3 (PPL00151-2a), pEGFP-N1-caspase-1 (PPL00392-2e), and mCherry-C1-ASC (PPL01752-2b) were bought from Public Protein/Plasmid Library (PPL, Nanjing, China). LY294002 (LY, T2008), compound C (CC, T6146), SCH772984 (SCH, T606), SBI-0206925 (SBI, T2128), and hydroxychloroquine (HCQ, T0951) were obtained from Topscience Co., Ltd. (Shanghai, China). Antibodies against LC3B (PM036) and Caspase-9 (0039) were purchased from MBL medical& biological laboratories Co., Ltd., (Nagoya, Japan). Antibodies, including p-AMPK (50071), AMPK (5832), p-mTOR (5536), mTOR (2983), p-Raf (2696), Raf (9433), p-MEK (9154), MEK (9122), p-ERK (4370), ERK (4695), p-ULK1 (Ser555, 5869), and p-ULK1 (Ser757, 6888), Bax (14796), Bcl-2 (3498), and IL-1β (12242), were purchased from Cell Signaling Technologies Inc (CST, Beverly, MA, USA). Antibodies against NLRP3 (sc-134306), caspase-1 (sc-56036), ASC (sc-514414), and β-actin (sc-8432) were bought from Santa Cruz Biotechnology (CA, USA). ULK1 (T56902S) was purchased from Abmart (Shanghai, China). Anti-CD45 (ab-208022) was bought from Abcam (Cambridge, MA, USA). Anti-TREM2 (29208) was purchased from Signalway Antibody (Maryland, USA). Anti-GFAP (bs-0199R) was obtained from Bioss (Beijing, China). All antibodies were commercially sourced and their specificity was validated by the suppliers.

### Preparation of fractions with different polarities from PCP

In brief, PCP leaves, stems, or flowers were smashed into crude powder and extracted with 70% ethanol using the refluxing method. Then, the extracted solutions were concentrated by rotary evaporation at 60 °C under vacuum conditions. The dried extract was resuspended with water and partitioned with petroleum ether (1:1 vol/vol) three times to obtain the PCP petroleum ether fraction (PF). The remaining water solution was then partitioned with ethyl acetate (1:1 vol/vol) three times to obtain the PCP ethyl acetate fraction (EF). The remaining water layer was then further partitioned with n-butanol (1:1 vol/vol) three times and dried to give the PCP n-butanol fraction (NF) and water fraction (WF), respectively. Finally, the WF, NF, EF, and PF of PCP were subjected to UHPLC-DAD-TOF/MS analysis.

### Cell culture

BV-2 cells and PC-12 cells were purchased from American Type Culture Collection (ATCC) (Rockville, MD, USA). Primary mouse microglia (CP-M110) and hippocampal neurons (CP-M107) were purchased from Procell Life Science & Technology Co. Ltd. (Wuhan, China). All cells were validated by the suppliers. BV-2 cells were cultured in RPMI 1640 medium supplemented with 20% fetal bovine serum (FBS), 50 U/mL penicillin, and 50 μg/mL streptomycin. PC-12 cells were maintained in DMEM supplemented with 10% HS, 5% FBS, and 1% PS. Primary mouse microglia were cultured in completed DM/F12 medium (CM-M110, Procell) supplemented with FBS, EGF, bFGF, and PS. Primary mouse hippocampal neurons were cultured in Neurobasal-A medium (CM-M107, Procell) composed of B-27 supplement and PS. Wild-type (WT) Atg7 and Atg7-deficient MEF cells were provided as generous gifts from Masaaki Komatsu (Juntendo University, School of Medicine, Tokyo, Japan) and were cultured in DMEM with 10% FBS, and 1% PS. All cells were maintained in a 5% humidified CO_2_ incubator at 37 °C.

### Isolation and identification of TA, TB, PHG, and PGHG

The EF of PCP flowers with potential autophagic effect was further isolated to obtain single compounds. In this experiment, an open column filled with silica gel was applied to preliminarily separate PCP-EF using petroleum ether, ethyl acetate, and methanol as an elution system, and a total of 10 fractions were collected. After the measurement of the autophagic effect in BV-2 cells, the seventh fraction (F7) with potent autophagic induction was further isolated by preparative high-performance liquid chromatography (Pre-HPLC) with the mobile phase consisting of acetonitrile and water.

All PCP derived fractions and compounds were analyzed using UHPLC (Agilent Technologies 1290 Series) equipped with time-of-flight (TOF) MS (Agilent Technologies 6230) with a jet stream ion source in negative ion mode and separated on an Agilent Zorbax Eclipse Plus C18 column with a particle size of 1.8 μm (flow rate: 0.35 mL/min). The elution program was set as follows: mobile phase A (0.1% formic acid in water) and mobile phase B (0.1% formic acid in ACN): 0–5 min, 5–50% B; 5–10 min, 50–100% B; 10–12 min, 100% B; 12–15 min, 5% B. The mass spectrum (MS) data were acquired in the scan mode from m/z 100 to 1700 Da with 2.0 spectra/s, and further analyzed using Agilent MassHunter Workstation software B.01.03.

### Cytotoxicity assay

All tested PCP-derived fractions or compounds were dissolved in DMSO and stored at −20 °C until further use. The cell viability of BV-2 cells and PC-12 cells under the treatments was measured using a 3-(4,5-dimethyl-2-thiazolyl)-2,5-diphenyl-2-H-tetrazolium bromide (MTT) reagent [56]. In brief, cells seeded into 96-well plates were treated with the tested fractions or compounds at a series of concentrations for the indicated hours. After treatment, 10 μL of MTT solution (5 mg/mL) was added to the wells for 4 h, followed by the dissolution of formazan with DMSO. Then, the colorimetric reading was performed by a spectrophotometer at 570 nm. The percentage of cell viability was calculated according to the following formula: Cell viability (%) = Cell number _treated_/Cell number _DMSO control_ × 100. Data were obtained from three independent experiments.

### Quantification of the number of GFP-LC3 puncta per cell

Quantification of the number of GFP-LC3 puncta per cell in GFP-LC3 transiently transfected cells was performed as previously described [7]. In brief, cells were seeded on coverslips in 6-well plates. After 24 h, cells were transfected with pEGFP-LC3 by the Exfect® Transfection Reagent (Vazyme Biotech Co., Ltd., Nanjing, China) according to the manufacturer’s instructions. Then, cells were subjected to treatment for 24 h, followed by 4% paraformaldehyde (PFA) fixation. The slides were air-dried and mounted with FluorSave™ mounting media (Calbiochem, San Diego, CA, USA). Representative images of cells were captured by a Leica SP8 confocal microscope with a LAS X 3D Visualization (Leica Microsystems Inc., Wetzlar, Germany). The average number of GFP-LC3 puncta per cell was calculated by ImageJ software (ImageJ 1.46r; National Institutes of Health, Bethesda, MD, USA). The color images were converted to grayscale images by auto-thresholding. Then GFP-LC3 punctated dots were determined in triplicates by counting a total of more than 30 cells. The criteria were as follows: particles with a size that is larger than background pixilation but smaller than the average nuclear were selected for the analysis, while apoptotic or necrotic cells were excluded from the analysis.

### mRFP-GFP tandem fluorescent-tagged LC3 (tfLC3) detection

BV-2 cells transfected with the tfLC3 plasmid were seeded on coverslips in 6-well plates and treated with TA or rapamycin (Rap, a positive autophagy inducer) under the indicated concentrations. After treatment, the BV-2 cells were fixed with 4% PFA and washed with PBS three times. The slides were taken out for air drying and mounted with FluorSave™ mounting media. The GFP-LC3 and RFP-LC3 puncta formations were examined by a Leica SP8 confocal microscope with a LAS X 3D Visualization (Leica Microsystems Inc., Wetzlar, Germany). The autophagy flux was evaluated by calculating the ratio of GFP/RFP fluorescence intensity by ImageJ software. A total of 300 cells from three randomly selected fields were scored [55].

### Transmission electron microscopy

BV-2 cells were treated with TA or Rap for 24 h. Then, cells were collected and cut into small specimens (one dimension < 1 mm) and fixed in Trump’s fixative (Electron Microscopy Sciences) for 2 h at room temperature. The sections were then washed with 0.1 M cacodylate buffer and postfixed in 1% osmium tetroxide, which was followed by dehydration in ethanol and embedment using the EMbed 812 kit (Electron Microscopy Sciences). Images were captured by a transmission electron microscope (JEM-1400FLASH). Autophagic vacuole is characterized by double-membrane structures surrounding the organelles or vesicles.

### Western blot

Cells or brain tissues were lysed by 1× RIPA lysis buffer (CST, Beverly, MA, USA) according to the manufacturer’s instructions, and the protein concentration was determined using the Bradford reagent (Bio-Rad, Hercules, CA, USA). Equal amounts of protein (30–50 μg/well) were loaded for sodium dodecyl sulfate-polyacrylamide gel electrophoresis (SDS-PAGE) analysis. The separated proteins on the gel were then transferred onto polyvinylidene fluoride (PVDF) membranes. After blockage with 5% non-fat dried milk for 1 h, the membranes were incubated with the primary antibodies overnight at 4 °C, which was followed by incubation with horseradish peroxidase (HRP)-conjugated secondary antibodies for 1 h. Then, the protein bands were revealed by the ultra-ECL Western Blotting Detection Reagent (4A Biotech Co., Ltd., China) and detected by the ChemiDoc MP Imaging System (Bio-Rad, Hercules, CA, USA). The protein expressions were quantified by calculating the band intensity using Image J software, and the relative expressions of the proteins of interest to the corresponding β-actin were obtained.

### Preparation of Aβ

A total of 5 mg of Aβ(1–42) was dissolved in 2 mL of hexafluoroisopropanol (HFIP) solution and then subjected to sonication for 15 min. The HFIP solution with Aβ peptide was aliquoted into 1.5-mL tubes (100 μL per tube), which were dried under a stream of nitrogen gas to produce a peptide film. The Aβ(1–42) peptide film was redissolved in 10 μL of DMSO and diluted with 490 μL culture medium to the final concentration for the in vitro experiments.

### The co-localization of LC3 with ASC

BV-2 cells seeded on confocal petri dishes were transiently transfected with pmCherry-C1-ASC and GFP-LC3 plasmids for 24 h, followed by treatment with Aβ(1–42) in the presence or absence of TA for 24 h. After treatment, BV-2 cells were fixed with 4% PFA and washed with PBS three times. The slides were taken out for air drying and mounted with FluorSave™ mounting media. Representative images of cells with pmCherry-C1-ASC and GFP-LC3 were captured using a Leica SP8 confocal microscope with a LAS X 3D Visualization (Leica Microsystems Inc., Wetzlar, Germany), and the co-localization of ASC and LC3 were revealed by the yellow spot.

### Hoechst 33342 and PI staining

Cell death of PC-12 cells was examined by Hoechst 33342 and PI staining as previously described [38]. Briefly, after treatment, PC-12 cells were fixed by 4% PFA and followed by the PBS wash three times. Then PC-12 cells were subjected to staining with 5 mg/L Hoechst 33342 and 5 mg/L PI solution for 10 min. After that, the slides were taken out to air-dry and mounted with FluorSave™ mounting media (Calbiochem, San Diego, CA, USA). Representative images of cells showing the blue and red signals were captured and merged by a fluorescence microscope. The cell death was analyzed by calculating the percentage of cells with PI signal to cells with Hoechst signal.

### Flow cytometry

Cell apoptosis of PC-12 cells was measured by flow cytometry using the Annexin V-FITC/PI staining kit (BD Biosciences, San Jose, CA, USA). In brief, after treatment, PC-12 cells were trypsined, collected, and centrifuged at a speed of 2000 rpm for 5 min. The supernatant was then removed, and the cell pellet was resuspended with 500 μL of 1× Annexin V solution. A mixture solution containing FITC and PI reagents was added to 1× Annexin V solution and incubated for 15 min in the dark. After incubation, cell apoptosis was analyzed by a Flow Cytometer (BD Biosciences, San Jose, CA, USA) according to the manufacturer’s instructions. Data acquisition and analysis were performed by FlowJo software (ACEA Biosciences).

### C. elegans strain and maintenance conditions

DA2123, adIs 2122[lgg-1p::GFP::Lgg-1+rol-6(su1006)] was obtained from the Department of Physiology, Tokyo Women’s Medical University School of Medicine (Tokyo, Japan). N2 (WT); BC12921, sIs10729 [rCes T12G3.1::GFP + pCeh361]; CL2331, dvIs37 [myo-3p::GFP::A-Beta (3-42) + rol-6(su1006)]; CL4176 [(pAF29) myo-3p::Aβ(1–42) + (pRF4) rol-6 (su1006)]; and BR5270, byIs161 [rab-3p::F3(delta)K280 + myo-2p::mCherry] were obtained from the Caenorhabditis Genetics Center (CGC), University of Minnesota (Minneapolis, MN, USA). All strains maintained on NGM plates were fed with *Escherichia coli* OP50 at 20 °C unless otherwise indicated.

### Measurement of p62 protein accumulation in the BC12921 strain

Measurement of the P62 protein accumulation was performed in the BC12921 strain as previously described [62]. In brief, the worms seeded in 96-well plates containing 100 μL of M9 buffer were pretreated with or without TA or Rap for 7 days. After treatment, representative images were captured, and the fluorescence intensities in the body of worms representing the expression of p62 were measured using a fluorescence microscope (Leica DM6B, Leica Microsystems GmbH, Germany) at excitation and emission wavelengths of 485 and 535 nm, respectively.

### Quantitative reverse transcription PCR

After treatment, the total RNA of BC12921 worms was extracted using the Trizol reagent. The purity and concentration of mRNA were determined by sepharose gel and a UV spectrophotometer, respectively. The reverse transcription to generate cDNA and the PCR reaction was conducted according to the manufacturerʼs protocol using the Prime Script RT reagent kit (TransGen Biotech, Beijing, China). The primers of p62 used were as follows: forward: AGTCACAATCCGCAATCGGT; reverse: AATCAGCCGATGGGAACAGT.

### Analysis of GFP::LGG-1 puncta in the DA2123 strain

Lgg-1 and lgg-2 are two Atg8 homologues in *C. elegans*. In this study, the autophagy effect of TA in *C. elegans* was also monitored by counting the number of GFP-positive LGG-1/Atg8 puncta in the DA2123 strain. In brief, worms were maintained at 20 °C and treated with or without TA or Rap on the day after hatching for 48 h. After treatment, the worms were mounted on a 2% agarose pad containing 0.1% NaN3 and GFP::LGG-1/Atg8 puncta were captured under the fluorescence microscope. The total number of GFP::LGG-1/Atg8 puncta in the same size area from 10 to 20 worms was counted by ImageJ software [53].

### Assay of food-sensing behavior in the BR5270 strain

The food-sensing behavior of *C. elegans* was assayed as previously described [50]. In brief, *C. elegans* N2 and BR5270 were seeded onto the 9 cm diameter Petri dish and spread with *E. coli* OP50 overnight in a ring with an inner diameter of 1 cm and an outer diameter of 8 cm. After treatment, the nematodes (*n* = 30–40) were washed with M9 buffer and released to the center of the NGM agar plate spotted with or without *E. coli* OP50 lawn. After five minutes, the body bends of each nematode were calculated for 1 min in the presence or absence of food. The slowing response was then obtained according to the following formula: Slowing rate = (*N*_without food_ – *N*_with food_)/*N*_without food_. N represents the total number of body bends.

### Measurement of Aβ(3–42) aggregation in the CL2331 strain

Measurement of the Aβ(3–42) aggregation was performed in the CL2331 strain, which temperature-sensitively expresses human Aβ(3–42) conjugated with GFP in the body wall muscle. In brief, the worms were treated with or without TA and cultured at 23 °C to induce Aβ(3–42) aggregation until day 2 of adulthood. After treatment, the worms were mounted on a glass slide containing 0.1% NaN3. Representative images were captured by a fluorescent microscope (Leica DM6B, Leica Microsystems GmbH, Germany), and the accumulation of amyloid deposits in the CL2331 worms was counted in the anterior area of worms.

### Paralysis assay in the CL4176 strain

A paralysis assay was performed in Aβ(1–42) transgenic *C. elegans* CL4176 as previously described [43]. In brief, the CL4176 strain expressing a heat-inducible human Aβ(1–42) transgene in muscle cells was treated with or without TA at 16 °C for 48 h. The plates were then transferred to 25 °C for the stimulation of Aβ expression and aggregation. The paralysis of worms was scored if worms exhibited “halos” of cleared bacteria around their heads or moved their head only or even did not move at all. The paralysis assay result was obtained from more than three independent experiments.

### RNAi treatment in the CL4176 strain

To investigate whether the neuroprotective effect of TA in *C. elegans* is associated with autophagy induction, we employed RNAi bacteria to knock down autophagy-related genes, including *unc-51*, *bec-1*, and *vps-34*, in CL4176 worms. In brief, eggs from CL4176 worms grown on OP50 were collected by bleaching, washed three times in M9 buffer, and then hatched in M9 buffer for 18 h. The synchronized L1 worms were transferred onto NGM plates and fed with the control bacteria HT115 or RNAi bacteria expressing *unc-51*, *bec-1*, and *vps-34*, respectively. After TA treatment, the paralysis was evaluated as described above.

### APP/PS1 mice and drug administration

All animal care and experiments in this study were approved by the Institutional Animal Care and Use Committee of Southwest Medical University, Luzhou, China (No. 201903-262). APPswe/PSEN1dE9 (APP/PS1) double transgenic (Tg) mice on a C57/BL6 background were purchased from the Jackson Laboratory (Bar Harbor, ME, USA). The normal control (NC) mice were generated by mating APP/PS1 mice and C57/BL6 mice. All mice were maintained in a room at 23 °C under a 12 h light-dark cycle. Six-month-old APP/PS1 mice were used for the study and randomly allocated to the vehicle group and drug treatment groups (0.25, 0.5, and 1.0 mg/kg of TA). All mice (8 mice in each group) were subjected to intraperitoneal (I.P.) injection with an equal volume of TA or blank reagent solution once daily for 2 months. After treatment, a Morris water maze test was performed to evaluate the cognitive function of mice. Then, all mice were sacrificed and the brains were collected. Among them, the brains from 4 mice were fixed in 4% PFA for immunohistochemistry and immunofluorescent staining, and the brains from 4 mice were frozen at − 80 °C for Western blotting analysis and ELISA.

### Morris water maze test

The cognitive function of mice was evaluated using a Morris water maze (MWM) test to detect the acquisition and retention of spatial memory as previously described [45]. The MWM system (Shanghai XinRuan Information Technology Co., Ltd., Shanghai, China) was equipped with a circular black pool (diameter 120 cm) and a VisuTrack Rodent Behavior Analysis software. An escape platform (5 cm in diameter) was located 1 cm below the water surface. Each mouse was trained for 6 consecutive days as it entered the water from the four quadrants and was given 60 seconds to swim onto the platform and stay on it for seconds. Then, the escape latency, the ratio of time spent in the target quadrant to the total time, the number of mice crossing the platform, and the average swimming speed were evaluated and recorded by the VisuTrack Rodent Behavior Analysis System.

### Immunohistochemistry

The expression of Aβ, NeuN, Bax, and NLRP3 in mouse brain tissue was detected using an immunohistochemical method as previously described [46]. In brief, the paraffinized sections were deparaffinized and rehydrated. Then, the sections were transferred into a container and covered with citrate buffer (pH = 6.0) and heated in a microwave for 10 min. The cooled slides were incubated with the primary antibodies overnight at 4 °C, which was followed by a wash and incubation with horseradish peroxidase (HRP)-conjugated goat anti-rabbit IgG antibody for 1 h at room temperature. The cells were developed with a 3,3′-diaminobenzidine (DAB) chromogen kit according to the manufacturer’s instructions. The representative images were captured by an optical microscope (Nikon, Japan), and the protein expressions were quantified by calculating the optical densities using ImageJ software (National Institutes of Health, Bethesda, MD, USA).

### Immunofluorescent staining

The expression of TUNEL, TREM2, GFAP, LC3, and CD45 in mouse brain tissue was detected using an immunofluorescence method [67]. Briefly, the sections were rinsed in PBS and blocked in 10% goat serum with 0.1% Triton X-100 for 1 h. Then, the sections were incubated with primary antibodies at 4 °C overnight, which was followed by a rinse in PBS. Then, the sections were incubated with fluorescent dye-conjugated secondary antibodies at room temperature for 2 h. After incubation, the sections were counterstained with DAPI and photographed by a Zeiss LSM 800 confocal microscope. The mean fluorescence intensity was measured by ImageJ software.

### Statistical analysis

All statistical data were obtained from three or more independent experiments. The data are presented as the mean ± standard deviation (SD) and analyzed by GraphPad Prism 5.0 statistical analytical software. One-way ANOVA analysis followed by a post-Tukey test was applied to compare the statistical difference among the groups. *P* < 0.05 was considered to have statistical significance.

## Results

### Characterization of PCP

PCP is a popular traditional herbal medicine that is widely distributed in the regions of China, including Sichuan, Guizhou, and Shanxi provinces. Among them, Gulin County in Luzhou City (Sichuan Province) is considered the geo-authentic habitat of PCP (Additional file 1: Fig. S1A). In this study, the characterization of PCP flower, leaf, and stem parts was performed by Agilent 6230 UHPLC-DAD-TOF/MS. The representative total ion chromatogram (TIC) in negative or positive ion mode and UV chromatogram monitored at 254 nm of PCP flower total ethanol extract (TEE) were shown in Additional file 1: Fig. S1B. The proposed compounds with the corresponding retention time and the measured accurate mass in negative or positive ion mode were listed in Additional file 1: Table S1. Among them, the compounds with a retention time of 7.862–8.017 min were identified as ellagitannin flavonoids, including TA, TB, PHG, PGHG, and other unidentified compounds, which are the main components in PCP [35, 49, 60]. In addition, the representative TIC in negative ion mode and UV chromatogram at 254 nm for PCP leaf and steam parts were displayed in Additional file 1: Fig. S1C, D, respectively, which were similar to those of PCP flowers.

### EF of PCP flower induces autophagy in BV-2 cells

After analysis of the components in each part of PCP, we found that the relative content of ellagitannin flavonoids including TA, TB, PHG, and PGHG in PCP flowers was more than those in PCP leaf or stem part (Additional file 1: Fig. S1; Table S2). To investigate which components in PCP-TEE activate autophagy in BV-2 cells, the TEE of the PCP flower, leaf, or steam was further isolated using reagents with different polarities, including water, n-Butanol, ethyl acetate, and petroleum ether. Finally, a total of 4 fractions, including water fraction (WF), n-butanol fraction (NF), ethyl acetate fraction (EF), and petroleum ether fraction (PF), were obtained. The analysis results showed that the ellagitannin flavonoids including PHG, TB, PGHG, and TA were mainly in the EF of PCP-TEE (Additional file 1: Fig. S1C, 1D, 2A; Tables S3-S5). Based on the above results, the PCP flower was selected for further bioactivity evaluation and isolation experiments. The safe treatment concentrations of WF, NF, EF, and PF in BV-2 cells were confirmed by MTT assay (Additional file 1: Fig. S2B). Then, the autophagic effect of WF, NF, EF, and PF in BV-2 cells was monitored by detecting the LC3 expression and counting the average number of GFP-LC3 puncta per cell. Among these fractions, PCP-EF showed the best effect in the increase of the conversion of the cytosolic form of LC3 (LC3-I) to LC3-phosphatidylethanolamine conjugate (LC3-II) and the average number of GFP-LC3 puncta per cell **(**Additional file 1: Fig. S2C, D). Taken together, the ellagitannin flavonoids, including TA, TB, PHG, and PHGH, are mainly in EF and are responsible for the activation of autophagy in BV-2 cells.

### Isolation, identification, and elucidation of TA, TB, PHG, and PHGH

The EF of PCP with potent autophagy was further isolated by column chromatography, and, ultimately, a total of nine fractions were generated. After analysis by Agilent 6230 UHPLC-DAD-TOF/MS (Additional file 1: Fig. S3A), the relative quantification results of TA, TB, PHG, and PHGH in F1–F9 showed that the ellagitannin flavonoids in F7 were higher than those in the other fractions (Additional file 1: Table S6). Then, the autophagic effect of these fractions in BV-2 cells was measured. As shown in Additional file 1: Fig. S3B, C, F7 significantly improved the average number of GFP-LC3 puncta per cell and the ratio of LC3-II/LC3-I. Through further isolation of PHG in F4 and TA, TB, and PHGH in F7 by Pre-HPLC equipped with a semi-preparative C18 column (254 × 10 mm, Thermo Fisher), 4 single peaks were collected for analysis using UHPLC-DAD-TOF/MS. The representative TICs and HPLC-UV chromatograms were displayed in Additional file 1: Fig. S4A and the mass spectra indicated that the measured accurate mass of TA, TB, PHG, and PHGH was 873.1534, 721.1451, 719.1322, and 871.1371, respectively (Additional file 1: Fig. S4B) [33, 35]. In addition, the ^1^H- and ^13^C- nuclear magnetic resonance (NMR) spectra of TA were shown in Additional file 1: Fig. S4C. After the MTT assay (Additional file 1: Fig. S5A), the protein expression of LC3 and the average number of GFP-LC3 puncta per cell were measured. As shown in Additional file 1: Fig. S5B, C, TA, TB, PHG, and PHGH significantly improved the ratio of LC3-II/LC3-I and the average number of GFP-LC3 puncta per cell. Among these, TA displayed the highest potency in autophagy induction in BV-2 cells. Therefore, TA was selected for further in vitro and in vivo experiments.

### TA induces autophagy flux in BV-2 and primary microglial cells

**Fig. 1:**
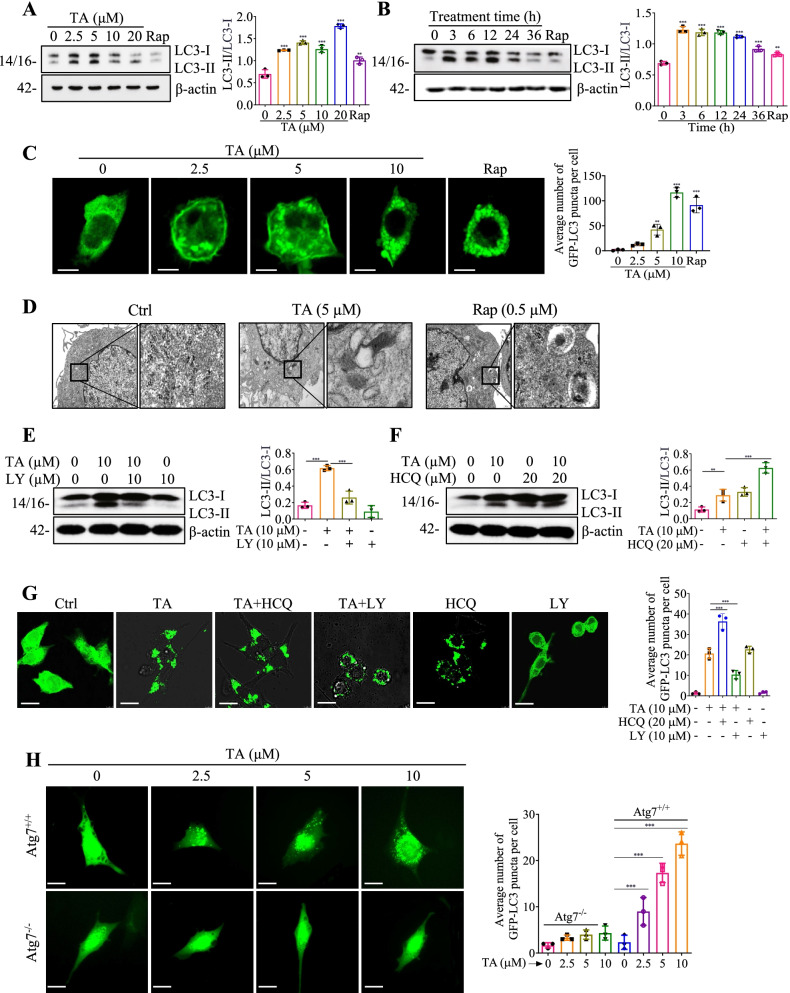
TA induces autophagy flux in microglial cells. **A**, **B** LC3 expressions of BV-2 cells treated with the indicated concentration of TA for 24 h or treated with 10 μM of TA for the indicated hours, and 0.5 μM rapamycin (Rap) was set as a positive autophagy inducer. Bar charts indicate the ratios of LC3-II/LC3-I. **C** Representative fluorescence images of mouse primary microglial cells transfected with GFP-LC3 plasmid and treated with TA or Rap for 24 h were captured. Magnification, × 63; scale bar: 25 μm. **D** Representative electron micrographs showing the ultrastructures of BV-2 cells which were treated with TA (5 μM), or Rap (0.5 μM) for 24 h. Left: Magnification: × 10000, scale bar: 2 μm; Right: Magnification: × 250000, scale bar: 500 nm. **E**, **F** LC3 expressions of BV-2 cells were pretreated with LY (10 μM) or HCQ (20 μM) for 1 h, followed by treatment with TA (10 μM) for an additional 24 h. Bar charts indicate the ratios of LC3-II/LC3-I in BV-2 cells. **G** Representative fluorescence images of BV-2 cells transfected with GFP-LC3 plasmid and pretreated with LY (10 μM) or HCQ (20 μM) for 1 h, followed by treatment with TA (10 μM) for an additional 24 h. Magnification, × 40; scale bar: 50 μm. The bar chart represents the average number of GFP-LC3 puncta per cell. **H** Representative fluorescence images of Atg7^+/+^ and Atg7^−/−^ MEF cells transfected with GFP-LC3 plasmid and treated with 2.5, 5, and 10 μM of TA for 24 h were captured. Magnification, × 40; scale bar: 50 μm. The bar chart represents the average number of GFP-LC3 puncta per cell. Error bars, S.D. *p ≤ 0.05; **p ≤ 0.01; ***p ≤ 0.001, *n* = 3. (One-way ANOVA with Tukey-corrected post hoc *t*-test for multiple comparisons was applied for comparison between groups). The full-length blots are presented in Additional file 1: Fig. S19

In this study, the autophagy induction effects of TA in BV-2 and mouse primary microglia cells were measured. Figure 1 A and B showed that TA significantly improved the ratio of LC3-II/LC3-I in BV-2 cells in a dose- and time-dependent manner. In addition, TA significantly increased the average number of GFP-LC3 puncta in mouse primary microglia (Fig. 1C) and BV-2 cells (Additional file 1: Fig. S6A). The autophagy flux was monitored in tfLC3 transiently transfected BV-2 cells and stable RFP-GFP-LC3 U87 cells, the results showed that TA dose-dependently decreased the ratio of GFP/RFP (Additional file 1: Fig. S6B; Video S1), suggesting that the GFP-LC3 puncta were degraded by TA. In addition, the electron microscopy images displayed that TA or Rap significantly increased the number of autophagosome-like organelles (Fig. 1D). Moreover, we employed LY, an early-stage autophagy inhibitor that inhibits the formation of autophagophore, and HCQ, an inhibitor that suppresses the normal function of lysosomes, to further investigate the early and later stages of TA-induced autophagy flux [29]. As shown in Fig. 1E, F, LY remarkably reduced the TA-induced conversion of LC3-I to LC3-II and the average number of GFP-LC3 puncta per cell. On the contrary, HCQ further promoted the conversion of LC3-I to LC3-II and increased the average number of GFP-LC3 puncta which were induced by TA (Fig. 1G). Taken together, the above data suggest that TA induces autophagy flux in microglial cells.

### TA induces autophagy via Atg7 in BV-2 cells

Emerging evidence indicates that LC3-I is activated by Atg7 and transferred to Atg3 upon autophagy induction. LC3-I is then conjugated to phosphatidylethanolamine (PE) and converted into LC-3-II, an autophagy inducer marker protein located on the membrane of autophagosomes [24]. To examine the role of Atg7 in TA-induced autophagy, wild-type (WT) mouse embryonic fibroblast (MEF) cells and Atg7-deficient MEF cells were applied in the current study. The safe concentrations of TA in Atg7^−/−^ and Atg7^+/+^ MEF cells were confirmed by MTT assay (Additional file 1: Fig. S6C). Then, the average number of GFP-LC3 puncta per cell and the protein expression of LsC3 were determined. As shown in Fig. 1H and Additional file 1: Fig. S6D, TA significantly increased the ratio of LC3-II/LC3-I and GFP-LC3 puncta formation in WT MEF cells but not in Atg7-deficient MEF cells. Taken together, these data suggest that TA activates autophagy via Atg7.

### TA induces autophagy mainly via the AMPK/ULK1 and Raf/MEK/ERK signaling pathways in BV-2 cells

**Fig. 2:**
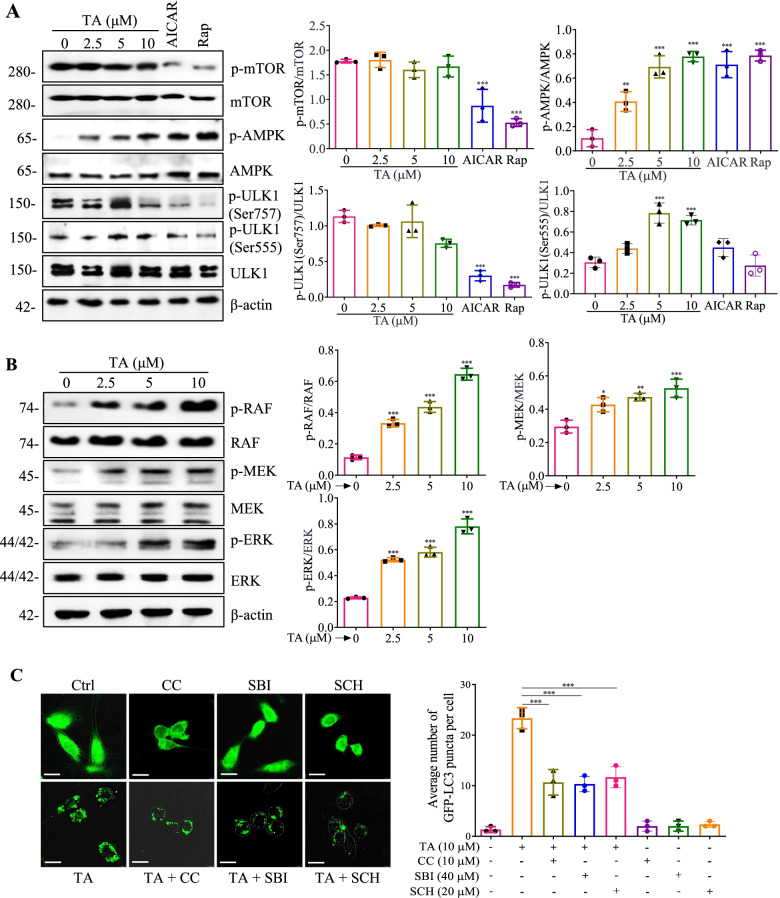
TA induces autophagy via activating the AMPK/ULK1 and Raf/MEK/ERK signaling pathways. **A** BV-2 cells were treated with 2.5, 5, and 10 μM of TA, AICAR (1 mM), or Rap (0.5 μM) for 24 h. Then protein expressions were detected using Western blot. Bar charts indicate the ratios of p-mTOR/mTOR, p-AMPK/AMPK, p-ULK1 (Ser757)/ULK1, and p-ULK1 (Ser555)/ULK1 in BV-2 cells. **B** BV-2 cells were treated with TA at indicated concentrations for 24 h. Then protein expressions were detected using Western blot. Bar charts indicate the ratios of p-Raf/Raf, p-MEK/MEK, and p-ERK/ERK in BV-2 cells. **C** Representative fluorescence images of BV-2 cells transfected with GFP-LC3 plasmid and pretreated with CC (10 μM) or SBI (40 μM) or SCH (20 μM) for 1 h, followed by treatment with TA (10 μM) for an additional 24 h were captured. Magnification, × 40; scale bar: 50 μm. Bar charts represent the average number of GFP-LC3 puncta per cell. Error bars, S.D. *p ≤ 0.05; **p ≤ 0.01; ***p ≤ 0.001, *n*=3. (One-way ANOVA with Tukey-corrected post hoc *t*-tests for multiple comparisons was applied for comparison between groups). The full-length blots are presented in Additional file 1: Fig. S20

The mechanistic target of rapamycin (mTOR), a serine/threonine kinase, mainly regulates cellular metabolism and growth. In addition, mTOR also plays a pivotal role in the regulation of autophagy, and PI3K/AKT/mTOR is the most important and classic pathway that negatively regulates autophagy [28]. In this study, the expression of mTOR in TA-treated BV-2 cells was investigated. As shown in Fig. 2A, AICAR and rapamycin significantly suppressed the protein expression of p-mTOR and its downstream protein, p-ULK1 (Ser757), while TA could not inhibit p-mTOR and p-ULK1 (Ser757). Therefore, TA induced autophagy independently of the mTOR signaling pathway. Emerging evidence indicates that AMP-activated protein kinase (AMPK), a key energy sensor that regulates cellular metabolism to maintain energy homeostasis, positively regulates autophagy [26]. Additionally, Atg1/ULK1 is a key regulator of autophagy from yeast to mammals, the latest works linking AMPK and ULK1 in mammalian cells have proven that AMPK directly activates autophagy via its interaction and phosphorylation of ULK1 at Ser555 [26]. Here, we demonstrated that TA remarkably upregulated p-AMPK and p-ULK1 (Ser555) in a dose-dependent manner (Fig. 2A). On the other hand, the Raf/MEK/ERK pathway serves as a key signal transducer of receptor tyrosine kinases and small GTPase is a tree-tiered kinase cascade, which plays a pivotal role in the regulation of cell survival, cell cycle progression, and differentiation [34]. Emerging evidence suggests that the Raf/MEK/ERK pathway also exerts a positive role in the regulation of autophagy [31]. In our current study, TA significantly improved the protein expression of phosphorylation of Raf, MEK, and ERK in a dose-dependent manner, suggesting that TA activated the Raf/MEK/ERK signaling pathway (Fig. 2B). Additionally, the regulative role of the AMPK/ULK1 and Raf/MEK/ERK signaling pathways in TA-induced autophagy was further validated by employing CC (an inhibitor of AMPK), SBI (an inhibitor of ULK1), and SCH (an inhibitor of ERK). As shown in Fig. 2C and Additional file 1: Fig. S7, CC, SBI, and SCH significantly decreased the average number of GFP-LC3 puncta per cell and the ratio of LC3-II/LC3-I. To explore whether there are relationships between AMPK/ULK1 and Raf/MEK/ERK or whether there are other pathways involved regarding TA-induced autophagy, we determined the activation of AMPK/ULK1 or Raf/MEK/ERK in BV-2 cells treated with TA in the presence of SCH or CC. As shown in Additional file 1: Fig. S8A, 8B, SCH significantly inhibited the protein expression of phosphorylation of AMPK and ULK1. Similarly, CC also suppressed the protein expression of phosphorylation of Raf, MEK, and ERK. In addition, Additional file 1: Fig. S8C showed that SCH and CC synergistically decreased the ratio of LC3-II/LC3-I in TA treated BV-2 cells. These data suggested that both AMPK/ULK1 and Raf/MEK/ERK might interact and synergistically contribute to TA-induced autophagy in BV-2 cells. Taken together, TA induces autophagy might mainly via activating the AMPK/ULK1 and Raf/MEK/ERK signaling pathways in microglial cells.

### TA promotes the autophagic degradation of NLRP3 inflammasome in Aβ(1–42)-induced BV-2 and primary microglial cells

**Fig. 3:**
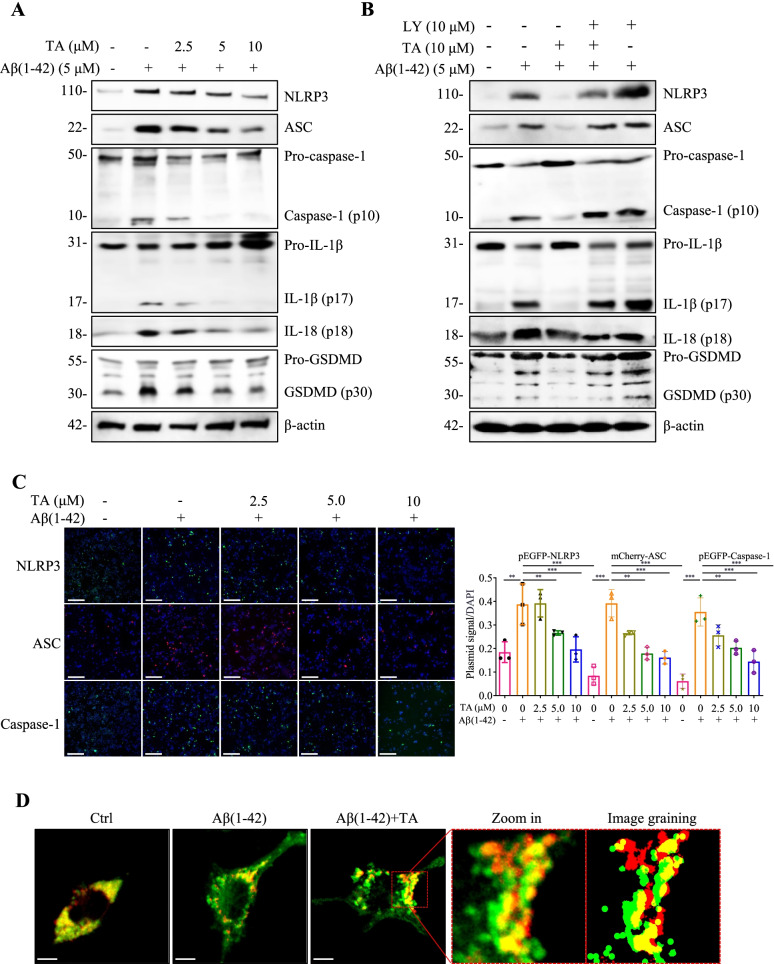
TA inhibits Aβ(1–42)-induced activation of the NLRP3 inflammasome via autophagy induction. **A** BV-2 cells were pretreated with 5 μM of Aβ(1–42) for 12 h, followed by treatment with TA at the indicated concentrations for an additional 24 h. Protein expressions were detected using Western blot. Bar charts indicate the ratios of NLRP3/β-actin, ASC/β-actin, caspase-1 (p10)/pro-caspase-1, IL-1β (p17)/pro-IL-1β, IL-18 (p18)/pro-IL-18, and GSDMD (p30)/pro-GSDMD in BV-2 cells. **B** BV-2 cells were pretreated with 5 μM of Aβ(1–42) for 12 h, followed by the treatment of TA (10 μM) in the presence or absence of LY (10 μM) for an additional 24 h. Protein expressions were detected using the Western blot. The bar chart indicates the ratios of NLRP3/β-actin, ASC/β-actin, caspase-1 (p10)/pro-caspase-1, IL-1β (p17)/pro-IL-1β, IL-18 (p18)/pro-IL-18, and GSDMD (p30)/pro-GSDMD in BV-2 cells. **C** Representative images of BV-2 cells transfected with pEGFP-NLRP3, mCherry-ASC, or pEGFP-caspase-1 plasmid for 18–24 h and treated with Aβ(1–42) in the presence or absence of TA at the indicated concentrations for 24h, followed by DAPI staining were captured. Magnification, × 10; scale bar: 100 μm. The bar chart indicates the percentage of BV-2 cells with NLRP3, ASC, or caspase-1 signal. **D** BV-2 cells seeded on confocal petri dishes were transiently transfected with pmCherry-C1-ASC and GFP-LC3 plasmid for 24 h, followed by treatment with 5 μM of Aβ(1–42) with or without TA (10 μM) for 24 h. After treatment, the BV-2 cells were fixed and examined by a Leica SP8 confocal microscope with a LAS X 3D Visualization (Leica Microsystems Inc., Wetzlar, Germany). Representative images of BV-2 cells with co-localization of pmCherry-C1-ASC and GFP-LC3 puncta were captured, and the image graining was performed by the Leica SP8 processor. Magnification, × 63; scale bar: 25 μm. Error bars, S.D. *p ≤ 0.05; **p ≤ 0.01; ***p ≤ 0.001, *n*=6. (One-way ANOVA with Tukey-corrected post hoc *t*-tests for multiple comparisons was applied for comparison between groups). The full-length blots are presented in Additional file 1: Fig. S21, 22

Emerging evidence indicates that the NLRP3 inflammasome is involved in the innate immune response in neurodegenerative diseases [13]. Upon stimulation of misfolded proteins, such as Aβ and Tau, the NLRP3 inflammasome is activated and an amount of proinflammatory cytokines, such as IL-1β and IL-18, is released from the overactivated microglia. In this study, we firstly examined the effect of TA on microglial morphology and phagocytic capacity. Figure S9 showed that TA (2–10 μM) alone had no significant effect on the morphology and phagocytic capacity of BV-2 cells. Then we employed Aβ(1–42) to activate the NLRP3 inflammasome in BV-2 cells and investigated the inhibitory effect of TA on the NLRP3 inflammasome. As shown in Fig. 3A, C and additional file 1: Fig. S10A, Aβ(1–42) significantly activated the NLRP3 inflammasome as evidenced by the increased protein expressions of NLRP3, ASC, and the cleavage form of caspase-1, IL-1β, and IL-18, as well as the increased fluorescence intensities of NLRP3, ASC, and caspase-1. After treatment of TA, the NLRP3 inflammasome was dose-dependently inhibited. Additionally, TA could also dose-dependently decrease Aβ(1–42)-induced IL-1β secretion and NLRP3 expression in BV-2 cells and primary microglial cells (Additional file 1: Fig. S11, 12). Taken together, TA inhibits Aβ(1–42)-induced overactivation of the NLRP3 inflammasome and the levels of IL-1β and IL-18 in microglial cells.

Research recently reported that autophagy negatively regulates proinflammatory responses and downregulates IL-1β, IL-18, IL-6, TNF-α, and other proinflammatory cytokines in microglial cells [2]. The dysfunction or inhibition of autophagy will aggravate the inflammatory responses and the pathology of neurodegenerative diseases [6, 69]. To examine whether TA-induced autophagy is involved in the inhibition of the NLRP3 inflammasome, we employed LY to suppress TA-induced autophagy and detected the activation of the NLRP3 inflammasome and the level of IL-1β. As shown in Fig. 3B, LY significantly attenuated the inhibitory effect of TA on the protein expressions of NLRP3, ASC, and the cleavage forms of caspase 1, IL-1β, and GSDMD in Aβ(1–42)-induced BV-2 cells. In addition, the ELISA results indicated that LY partially abolished the inhibitive effect of TA on IL-1β secretion (Additional file 1: Fig. S11). Moreover, the confocal microscopy images displayed that TA induced the co-localization of GFP-LC3 puncta and ASC speck as revealed by the increased number of yellow spots in pmCherry-C1-ASC and GFP-LC3 co-transfected BV-2 cells (Fig. 3D), which directly demonstrated that TA inhibited the NLRP3 inflammasomes via an autophagic degradation mechanism. Taken together, TA inhibits the overactivation of NLRP3 inflammasome via autophagy induction in Aβ(1–42)-induced BV-2 cells.

**Fig. 4:**
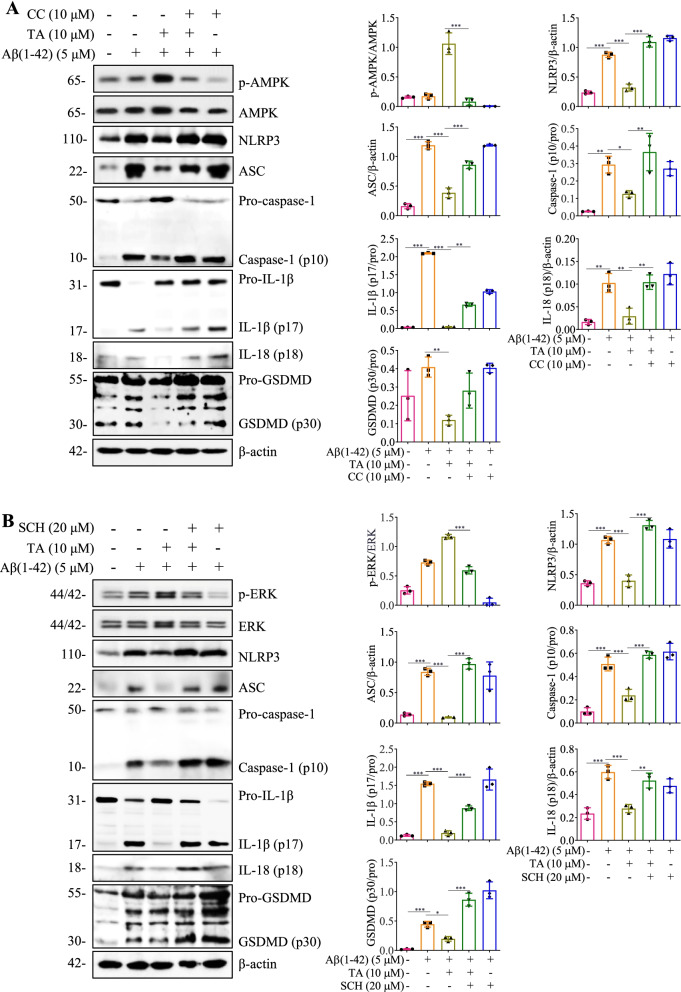
TA inhibits Aβ(1–42)-induced activation of the NLRP3 inflammasome through the AMPK/ULK1- and Raf/MEK/ERK-mediated autophagy. **A**, **B** BV-2 cells were pretreated with 5 μM of Aβ (1–42) for 12 h, followed by the treatment of TA (10 μM) in the presence or absence of CC (10 μM) or SCH (20 μM) for an additional 24 h. Then protein expressions were detected using Western blot. Bar charts indicate the ratios of NLRP3/β-actin, ASC/β-actin, caspase-1 (p10)/pro-caspase-1, IL-1β (p17)/pro-IL-1β, IL-18 (p18)/pro-IL-18, and GSDMD (p30)/pro-GSDMD in BV-2 cells. **B** Error bars, S.D. *p ≤ 0.05; **p ≤ 0.01; ***p ≤ 0.001, *n* = 3. (One-way ANOVA with Tukey-corrected post hoc *t*-tests for multiple comparisons was applied for comparison between groups). The full-length blots are presented in Additional file 1: Fig. S23

In addition to the blockage of autophagy flux, we also employed CC and SCH to inhibit the AMPK/ULK1 and Raf/MEK/ERK signaling pathways and then investigated the inhibitory effect of TA on the activation of the NLRP3 inflammasome. As shown in Fig. 4A, CC significantly decreased TA-induced AMPK phosphorylation and attenuated the inhibitory effect of TA on the protein expression of NLRP3, as well as the cleavage of caspase-1 and IL-1β. Additionally, SCH also remarkably decreased TA-induced ERK phosphorylation and turned over the inhibitory effect of TA on the activation of the NLRP3 inflammasome (Fig. 4B). Furthermore, both CC and SCH partially reversed the effect of TA on the decrease of IL-1β secretion in Aβ(1–42)-treated BV-2 cells (Additional file 1: Fig. S11). Taken together, these data suggested that TA inhibits the activation of NLRP3 inflammasome and decreased the levels of proinflammatory cytokines such as IL-1β and IL-18 via both AMPK/ULK1- and Raf/MEK/ERK-mediated autophagy in Aβ(1–42)-induced BV-2 cells.

### TA ameliorates the damage of PC-12 and primary neuronal cells

**Fig. 5:**
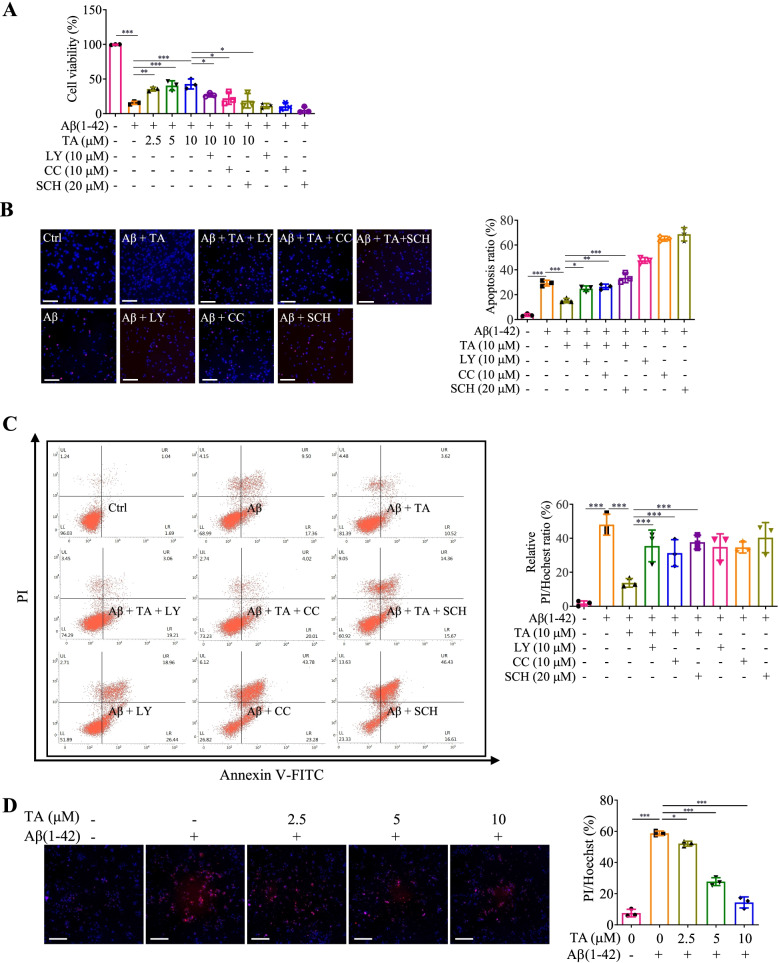
TA ameliorates neuronal damage induced by the Aβ(1–42)-induced activation of the NLRP3 inflammasome in microglial cells. BV-2 cells were treated with 5 μM of Aβ(1–42) for 24 h, followed by the treatment of TA (10 μM) in the presence or absence of inhibitors, including LY, CC, and SCH for an additional 24 h. The conditioned mediums were then transferred into PC-12 cells and incubated for 24 h. The cell viability of PC12 cells was then detected by (**A**) MTT assay, (**B**) Hoechst 33342/PI staining, and (**C**) flow cytometry methods. Bar charts indicate the cell viability, the PI/Hoechst ratio, and the apoptosis rate of PC-12 cells. The representative fluorescence images were captured by a fluorescence microscope. Magnification, × 20; scale bar: 100 μm. **D** Mouse primary microglial cells were treated with 5 μM of Aβ(1–42) for 24 h, followed by the treatment of TA at the indicated concentrations for 24 h. The conditioned mediums were then transferred into mouse primary hippocampal neurons and incubated for 24 h. The cell viability of mouse primary hippocampal neurons was then detected by Hoechst 33342/PI staining method. The representative fluorescence images were captured by a fluorescence microscope. Magnification, × 20; scale bar: 100 μm. The bar chart indicates the PI/Hoechst ratio of mouse primary hippocampal neurons. Error bars, S.D. *p ≤ 0.05; **p ≤ 0.01; ***p ≤ 0.001. *n* = 3. (One-way ANOVA with Tukey-corrected post hoc *t*-test for multiple comparisons was applied for comparison between groups)

Emerging evidence indicates that the production of proinflammatory cytokines from microglia results in neuronal damage and neurodegeneration [18]. Upon stimulation of Aβ(1–42), the proinflammatory cytokines, such as IL-6, IL-1β, and TNF-α, are released from BV-2 cells [44, 68]. In this study, the conditional medium was prepared through the incubation of BV-2 cells with Aβ alone or co-treated with TA in the presence or absence of inhibitors, including LY, CC, and SCH, for 24 h. Then, the conditional medium was transferred into PC-12 cells or mouse primary hippocampal neurons for further incubation. After 24 h, the viability of the PC-12 cells was measured by MTT, Hoechst33342/PI staining, and flow cytometry methods. As shown in Fig. 5A–C and Additional file 1: Fig. S13, TA dose-dependently increased the viability, as well as decreased the apoptosis ratio and the ratio of PI/Hochest of PC-12 cells. Meanwhile, TA also recovered the cell viability of mouse primary hippocampal neurons as evidenced by the decreased ratio of PI/Hochest of cells (Fig 5D). While the inhibitors, including LY, CC, and SCH, reversed the ameliorative effect of TA on the damage of PC-12 cells (Fig. 5A–C and Additional file 1: Fig. S13). On the other hand, we also found that TA significantly inhibited the fibrillization of Aβ (Additional file 1: Fig. S14A), and the bio-layer interferometry (BLI) analysis indicated that there was a good affinity between TA and Aβ (Additional file 1: Fig. S14B). Emerging evidence indicates that the overaccumulation of Aβ results in neuronal death [16]. In this study, we further investigated the inhibitory effect of TA on the cell death of mouse primary hippocampal neurons induced by Aβ(1–42). As shown in Additional file 1: Fig. S14C, TA significantly decreased the ratio of PI/Hochest in primary neurons, suggesting that TA could inhibit the damage of neurons induced by misfolded proteins such as Aβ. Increasing studies show that autophagy induction plays an important role in the inhibition of neuronal damage [54]. Therefore, we also detected the autophagy induction of TA in mouse primary hippocampal neurons, and Additional file 1: Fig. S14D showed that TA dose-dependently increased the average number of GFP-LC3 puncta per cell. Taken together, all these data demonstrated that TA ameliorates the neuronal damage induced by the overactivation of neuroinflammation and the overaccumulation of misfolded proteins such as Aβ might via autophagy induction.

### TA induces autophagy and exhibits neuroprotective effects in C. elegans

**Fig. 6:**
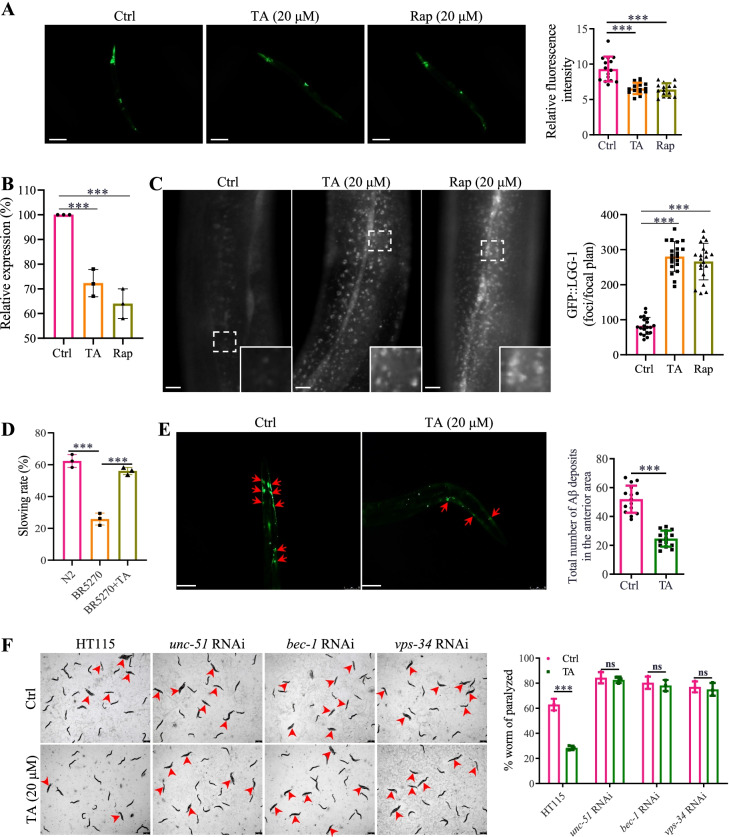
TA induces autophagy and exhibits a neuroprotective effect in *C. elegans*. **A** Representative images of BC12921 worms with GFP representing the expression of p62. Magnification, × 10; scale bar: 200 μm. The bar chart indicates the relative GFP intensity (*n* = 10). **B** The bar chart indicates the relative mRNA expression of p62 in worms (*n* = 10). **C** Representative images of DA2123 worms with GFP::LGG-1 puncta. Magnification, × 20; scale bar: 100 μm. Inset images displayed a higher magnification of the image in the dashed rectangle. The bar chart indicates the number of GFP::LGG-1 puncta in worms (*n* = 10). The results were pooled from three independent experiments. The full image of worms is presented in Additional file 1: Fig. S24. **D** The bar chart indicates the slowing rate of the N2 and BR5275 worms treated with or without TA (20 μM) (*n* = 60). **E** Representative images of CL2331 worms with GFP representing the amount of Aβ deposits. Magnification, × 10; scale bar: 200 μm. The bar chart indicates the number of Aβ deposits in the anterior area of worms (*n* = 10). **F** Representative images of CL4176 worms treated with TA (20 μM) in the presence or absence of *unc-51* RNAi, *bec-1* RNAi, or *vps-34* RNAi bacteria were captured, and red arrows indicated the paralyzed worms. Magnification, × 10; scale bar: 200 μm. The bar chart indicates the percentage of paralyzed worms (*n* = 60). Error bars, S.D. **p ≤ 0.01; ***p ≤ 0.001. (One-way ANOVA with Tukey-corrected post hoc *t*-tests for multiple comparisons was applied for comparison between groups)

Emerging evidence indicates the important role of autophagy in anti-aging and antioxidative effects to protect against aging-related diseases, such as AD [39, 41]. In this study, we examined the autophagy effect of TA in vivo by detecting the expression of p62 in the BC12921 strain and calculating the GFP-LGG-1 puncta in the DA2123 strain [25, 40]. As shown in Fig. 6A and B, TA and Rap significantly decreased the fluorescence intensity and reduced the relative mRNA expression of p62. In addition, TA and Rap increased the formation of GFP-LGG-1-positive puncta in worms (Fig. 6C). Therefore, TA was demonstrated to induce autophagy in *C. elegans.* To further investigate and confirm the neuroprotective effect of TA in multiple *C. elegans* models of AD, we first evaluated the food-sensing behavior in Tau transgenic BR5270 *C. elegans*. Figure 6D showed that TA significantly attenuated the food searching deficit in the BR5270 strain. In addition, the Aβ deposits in the TA-treated CL2331 strain were measured, and the images in Fig. 6E displayed that TA remarkably decreased the Aβ deposits in the anterior area of the worms. To investigate whether TA can improve Aβ(1–42)-induced behavior in vivo, the paralysis of worms in CL4176 nematodes expressing human Aβ peptide was investigated. As shown in Fig. 6F, TA exhibited a significant delay of Aβ(1–42)-induced paralysis in the CL4176 nematodes. However, after the knockdown of key autophagy-related genes, including *unc-51*, *bec-1*, and *vps-34*, using RNAi bacteria, the paralysis of TA-treated CL4176 nematodes was significantly increased, suggesting that TA inhibited the paralysis of CL4176 worms via autophagy induction. Taken together, TA induced autophagy, cleared Aβ, and improved behavioral performance in *C. elegans*.

### TA improves cognitive function and reduces the expression of Aβ and neuronal apoptosis in APP/SP1 mice

**Fig. 7:**
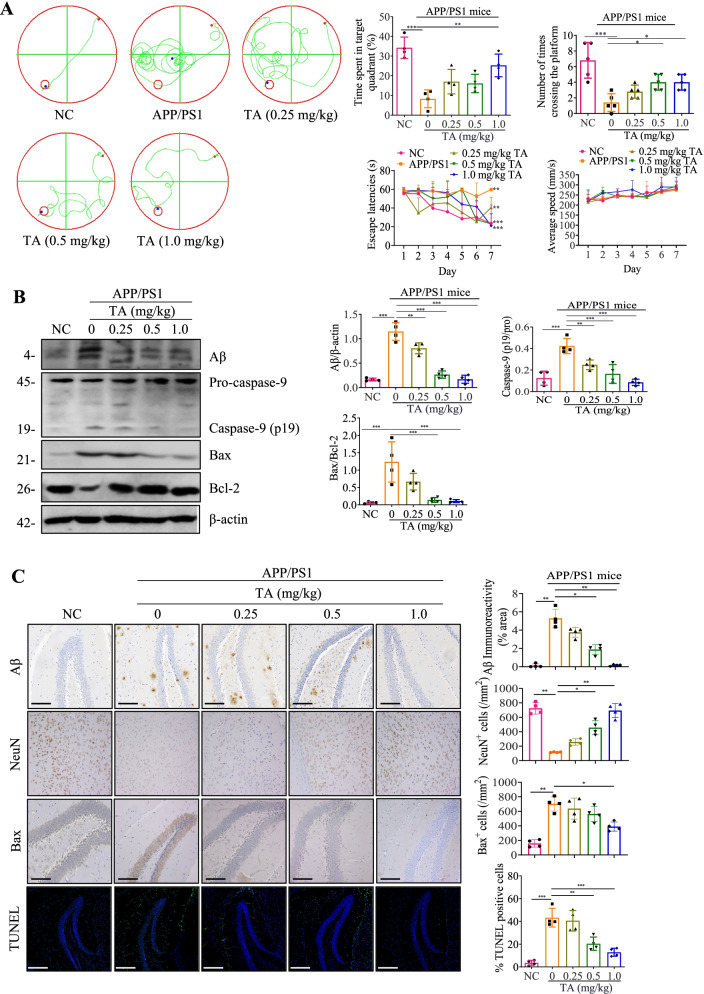
TA improves cognitive function and ameliorates the Aβ pathology and neuronal damage in APP/PS1 mice. **A** The cognitive function of NC and APP/PS1 mice was evaluated by the MWM test. Bar charts indicate the escape latencies, the ratio of time spent in the target quadrant/total time, the number of mice crossing the platform, and average swimming speed (*n* ≥ 4). **B** The Western blotting detection of the protein expressions of Aβ, caspase-9, Bax, and Bcl-2 in the brain tissues of NC and APP/PS1 mice. Bar charts indicate the ratios of Aβ/β-actin, caspase-9 (p19)/Pro, Bax/Bcl-2. The full-length blots are presented in Additional file 1: Fig. S25. **C** Representative immunohistochemistry and immunofluorescence staining images showing the expressions of Aβ, NeuN, Bax, and TUNEL-positive neurons in the hippocampus of NC and APP/PS1 mice. Magnification: × 20; scale bar: 100 μm. Bar charts indicate the quantification of Aβ, NeuN, Bax, and TUNEL-positive neurons (*n* = 4). Error bars, S.D. *p ≤ 0.05; **p ≤ 0.01; ***p ≤ 0.001, *n* = 4. (One-way ANOVA with Tukey-corrected post hoc *t*-tests for multiple comparisons was applied for comparison between groups)

There is a growing body of evidence showing the contributive role of NLRP3 inflammasome in the decline of cognitive function in AD [13, 14]. In recent years, increasing evidence indicates that the accumulation of Aβ fibrils induces neuronal death and accelerates brain degeneration [8]. In this study, we employed APP/PS1 mice to further investigate whether TA ameliorates cognitive impairment and inhibits the expression of Aβ and apoptosis protein in vivo. After intraperitoneal (I.P.) administration of TA for two months, there is no obvious difference found in weight, diet, hair, and skin of mice among the groups, suggesting the administration of TA in APP/PS1 mice is safe. The MWM test results showed that the cognitive impairment of APP/PS1 mice was significantly ameliorated by TA as demonstrated by the enhanced spatial learning and memory performance, including a decreased escape latency, the ratio of time spent in target quadrant, and the number of times crossing the platform (Fig. 7A). In addition, the Western blotting results indicated that TA could dose-dependently decrease the expression of Aβ and apoptosis-related proteins including caspase-9 and Bax/Bcl-2 in the brain of APP/PS1 mice (Fig. 7B). Furthermore, the immunohistochemistry and immunofluorescence results showed that TA ameliorated Aβ deposition in the brain, increased the number of NeuN-positive neurons, and inhibited the expression of Bax and TUNEL-positive neurons (Fig. 7C). Moreover, the ELISA result also showed that TA decreased the Aβ level in the brain of APP/PS1 mice (Additional file 1: Fig. S15). Therefore, the above data suggested that TA improved cognitive function and ameliorated Aβ pathology and neuronal damage in APP/PS1 mice.

### TA suppresses the NLRP3 inflammasome via the AMPK/ULK- and Raf/MEK/ERK-mediated autophagy in APP/PS1 mice

**Fig. 8:**
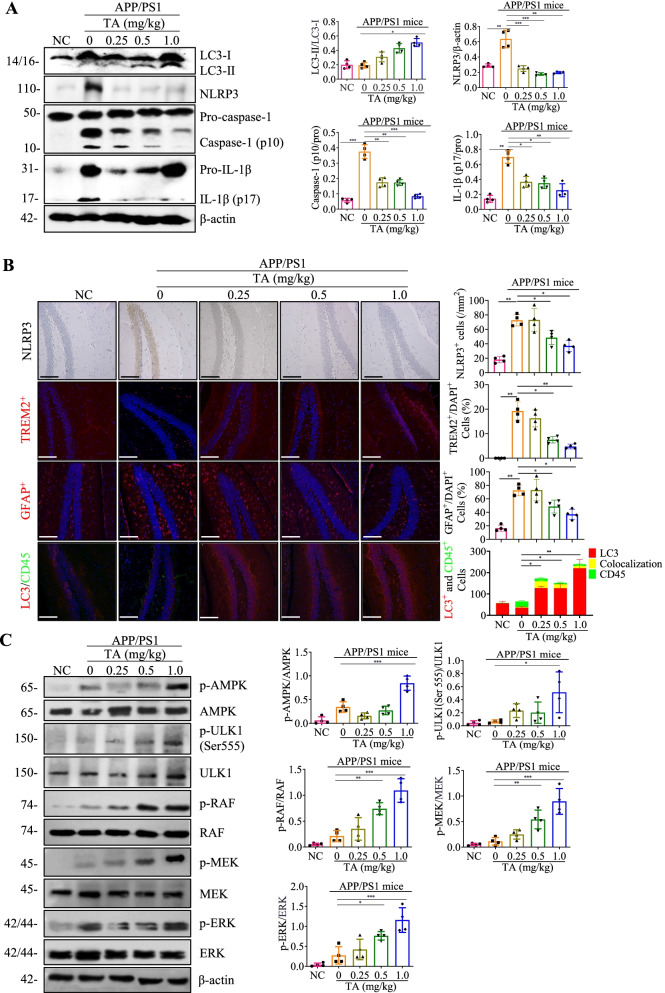
TA inhibits the activation of NLRP3 inflammasome might via the AMPK/ULK1- and Raf/MEK/ERK-mediated autophagy in APP/PS1 mice. **A** The Western blotting detection of the protein expressions of LC3, NLRP3, caspase 1, and IL-1β in the brain tissues of NC and APP/PS1 mice. The bar chart indicates the ratios of LC3-II/β-actin, NLRP3/β-actin, caspase-1 (p10)/Pro, and IL-1β (p17)/Pro. **B** Representative immunohistochemistry and immunofluorescence staining images showing the expressions of NLRP3, TREM2, GFAP, LC3, and CD45 in the hippocampus of NC and APP/PS1 mice. Magnification: × 20; scale bar: 100 μm. Bar charts indicate the quantifications of NLRP3 expression, and the cells with TREM2^+^/DAPI^+^, GFAP^+^/DAPI^+^, and LC3^+^, CD45^+^, and the colocalization of LC3^+^ with CD45^+^ (*n* = 4). **C** The Western blotting detection of the protein expressions of p-AMPK, AMPK, p-ULK1(555), ULK1, p-RAF, RAF, p-MEK, MEK, p-ERK, ERK, and β-actin in the brain tissues of NC and APP/PS1 mice. The bar chart indicates the ratios of p-AMPK/AMPK, p-ULK1(555)/ULK1, p-RAF/RAF, p-MEK/MEK, p-ERK/ERK. Error bars, S.D., *p ≤ 0.05, **p ≤ 0.01, ***p ≤ 0.001. The full-length blots are presented in Additional file 1: Fig. S26

In this study, we further investigated whether TA induces autophagy and inhibits the activation of the NLRP3 inflammasome in mice. The protein expression of LC3 and the key components of NLRP3 inflammasome in the brain tissue were detected by Western blotting, immunohistochemistry, and immunofluorescence methods. As shown in Fig. 8A, TA significantly improved the ratio of LC3-II/LC3-I and inhibited the activation of the NLRP3 inflammasome as evidenced by the decreased protein expression of NLRP3 and the cleavage forms of caspase-1 and IL-1β. In addition, the immunohistochemistry results showed that TA significantly inhibited the expression of NLRP3 in a dose fashion (Fig. 8B). Meanwhile, the neuroinflammatory indicators including TERM2 and GFAP detected by immunofluorescence were also dose-dependently inhibited by TA (Fig. 8B). To demonstrate that TA-induced autophagy is occurring specifically in microglial cells, the double immunostaining of LC3 and CD45 was performed using the immunofluorescence method. As shown in Fig. 8B, TA significantly decreased the number of spots with green fluorescence intensity (CD45) while increasing the red fluorescence intensity (LC3). Meanwhile, the colocalization of LC3 with CD45 was also increased by TA. Furthermore, the ELISA results also showed that TA decreased the level of IL-1β, IL-18, NLRP3, TERM2, and GFAP in the brain of APP/PS1 mice (Additional file 1: Fig. S15). Therefore, the above data suggested that TA inhibited the NLRP3 inflammasome-mediated inflammatory responses and the overactivation of microglia and astrocytes might be via autophagy induction. Moreover, we investigated whether TA could activate the AMPK/ULK1 and Raf/MEK/ERK signaling pathways, and the Western blotting results showed that TA significantly increased the protein expression of phosphorylation of AMPK, ULK1, Raf, MEK, and ERK (Fig. 8C). Finally, the brain-blood barrier (BBB) permeation of TA was determined by the UHPLC-DAD-TOF/MS instrument, and TA was detected successfully in the whole brain tissue of PCP-TEE-treated mice (Additional file 1: Fig. S16), suggesting that TA might be a BBB permeable compound. Taken together, TA is a potential natural compound that will be further developed into a novel candidate for AD in the future.

## Discussion

AD is characterized by deposits of extracellular Aβ plaques and intraneuronal NFTs. In addition, Aβ-induced NLRP3 inflammasome activation is recognized as a critical microglia sensor in the mediation of IL-1β and IL-18 secretion [52]. Emerging evidence has indicated that autophagy, a catabolic process, negatively regulates the NLRP3 inflammasome, which inhibits the proinflammatory responses in the brain of AD. Therefore, targeting the inhibition of NLRP3 inflammasome-mediated neuroinflammation by autophagy enhancers has become a promising strategy for the therapy of AD [38].

In our previous study, we identified that ellagitannin flavonoids were potent autophagy inducers in PCP. In this study, we confirmed the strongest autophagy induction effect of TA in BV-2 cells and further clarified the molecular mechanism of TA-induced autophagy. mTOR plays an important role in the regulation of autophagy. There are two distinct functional complexes of mTOR, including mTORC1 and mTORC2. During starvation, the activity of mTORC1 is inhibited and autophagy is activated to recycle intracellular constituents to provide energy for cell survival. mTORC1 is regulated by growth factors, such as insulin or insulin-like growth factor. The binding of insulin to the cell surface receptors activates the PI3KC1a complex, which mediates the conversion of PtdIns(4,5)P2 to generate PtdIns(3,4,5)P3. Stimulation of the PI3KC1a pathway recruits PDK1 and Akt to the plasma membrane, which then activates mTORC1 and inhibits autophagy. In addition, the activity of mTORC1 can also be regulated by changes in the energy state through the AMPK/TSC pathway. AMPK, a cellular energy sensor, can inhibit the activity of mTORC1 and positively regulate autophagy.

In this study, the protein expression of mTOR was investigated upon the treatment of TA in BV-2 cells. Figure 2A displayed that TA could not inhibit the protein expression of p-mTOR, suggesting TA induced autophagy independently of the mTOR signaling pathway, which was consistent with our previous study [42]. However, the protein expression of p-AMPK was significantly increased by TA. In addition, CC (the specific inhibitor of AMPK) inhibited TA-induced ratio of LC3-II/LC3-I and the average number of GFP-LC3 puncta per cell. ULK1/Atg1 is a serine/threonine protein kinase, and the activity of ULK1 is essential for autophagy initiation. Studies reported that autophagy can be directly induced via the activation of p-ULK1 at Ser317, Ser555, or Ser777 under the condition that p-AMPK is upregulated, or via the dephosphorylation of ULK1 at Ser757 if mTORC1 is down-regulated [11]. In this study, TA increased the protein expression of p-ULK1(Ser555) but did not affect the protein expression of p-ULK1(Ser757). In addition, SBI inhibited the protein expression of LC3-II and the number of GFP-LC3 puncta per cell in TA treated BV-2 cells. Taken together, TA induced autophagy via AMPK/ULK1 but was independent of the mTOR signaling pathway. Recent studies reported that the ERK signaling pathway positively regulates autophagy [36, 52]. Therefore, we also investigated the role of the Raf/MEK/ERK signaling pathway in the regulation of TA-induced autophagy. Our data here clearly indicated that TA activated the Raf/MEK/ERK signaling pathway, and SCH (an inhibitor of ERK) remarkably attenuated TA-induced autophagy. Moreover, emerging evidence indicates that there is an interplay between AMPK/ULK1 and Raf/MEK/ERK signaling pathways [32, 64]. To further explore the relationship between the AMPK/ULK1 and Raf/MEK/ERK signaling pathway, we investigated whether CC could inhibit the effect of TA on the activation of the Raf/MEK/ERK pathway. Meanwhile, we also investigated whether SCH could inhibit the effect of TA on the activation of the AMPK/ULK1 pathway. Finally, we found that CC or SCH significantly abolished the effect of TA on the activation of the Raf/MEK/ERK pathway or AMPK/ULK1 pathway. Meanwhile, the co-treatment of CC with SCH further inhibited the effect of TA on autophagy induction (Additional file 1: Fig. S8C). These data suggested that AMPK/ULK1 and Raf/MEK/ERK interacted and synergistically contributed to the regulation of TA-induced autophagy. Moreover, we also used the network pharmacology theory to explore the potential targets of TA. Through target prediction and protein interaction network research, combined with molecular docking simulation, we found that HSP90 was the key target that regulated the proteins involved in the AMPK/ULK1 and Raf/MEK/ERK signaling pathways. Meanwhile, the molecular docking showed that the binding score simulated by SYBLY of TA with HSP90AA1 was high to 9.91, suggesting that HSP90 might be the direct target for TA in the regulation of AMPK/ULK1 and Raf/MEK/ERK signaling pathways (Additional file 1: Fig. S17). In our future study, we will validate this prediction and deeply investigate the regulative effect of TA on HSP90.

Increasing evidence suggests that autophagy enhancers not only degrade extracellular Aβ but also inhibit Aβ-induced NLRP3 inflammasome activation and the release of proinflammatory cytokines [6]. The present study aimed to investigate whether TA could inhibit the Aβ-induced NLRP3 inflammasome in vitro and in vivo. We found that TA significantly inhibited the activation of the NLRP3 inflammasome, the levels of IL-1β and IL-18, and its induced damage to PC-12 cells. In addition, we have demonstrated that TA induces autophagy via activating AMPK/ULK1 and Raf/MEK/ERK signaling pathways in BV-2 cells, and CC and SCH significantly inhibited TA-induced autophagy. Therefore, we employed LY, CC, and SCH to investigate whether TA inhibited the activation of the NLRP3 inflammasome via AMPK/ULK1- and Raf/MEK/ERK-mediated autophagy. The results showed that the inhibitory effect of TA on the activation of the NLRP3 inflammasome was partially abolished by LY, CC, and SCH. Emerging evidence indicates TLR4 signaling participates in the inflammatory response and NLRP3 activation in microglia [61]. Thus, the present study further investigated whether TA could inhibit the NLRP3 inflammasome via the inhibition of TLR4. Finally, we found that TAK-242, a positive inhibitor of TLR4, significantly inhibited TLR4 expression and the protein expression of NRLP3, ASC, and the active forms of caspase-1, IL-1β, IL-18, and GSDMD. In contrast, TA could not inhibit the expression of TLR4 (Additional file 1: Fig. S18). Although our current study mainly focused on the inhibitory effect of TA on the NLRP3 inflammasome and determined the levels of IL-1β and IL-18 in vitro and in vivo, we will investigate whether TA exerts an anti-inflammatory effect via other mechanisms and inhibits the levels of IL-8, IL-6, TNF-α, other common proinflammatory cytokines. Based on the above findings, we concluded that TA inhibited the activation of the NLRP3 inflammasome via an autophagic degradation mechanism in microglia. In addition, neurons could be damaged by the accumulated misfolded proteins or overproduction of proinflammatory cytokines, while autophagy plays a pivotal role in the degradation of misfolded proteins and the inhibition of the inflammatory response. The present study demonstrated that TA acting as a potent autophagy enhancer could improve the viability of neuronal cells under the conditions of proinflammatory response or Aβ-induced damage.

In in vivo experiments, TA was demonstrated to decrease p62 expression and increase the formation of GFP-LGG-1-positive puncta, as well as improve food-searching deficits, decrease Aβ deposits, and delay paralysis in *C. elegans*. Moreover, the effect of TA on the delay of paralysis is closely associated with the regulation of key autophagy-related genes, including *unc-51*, *bec-1*, and *vps-34.* In APP/PS1 mice, TA significantly improved cognitive function, ameliorated Aβ pathology, as well as inhibited neuronal apoptosis and NLRP3 inflammasome-mediated proinflammatory responses.

**Fig. 9:**
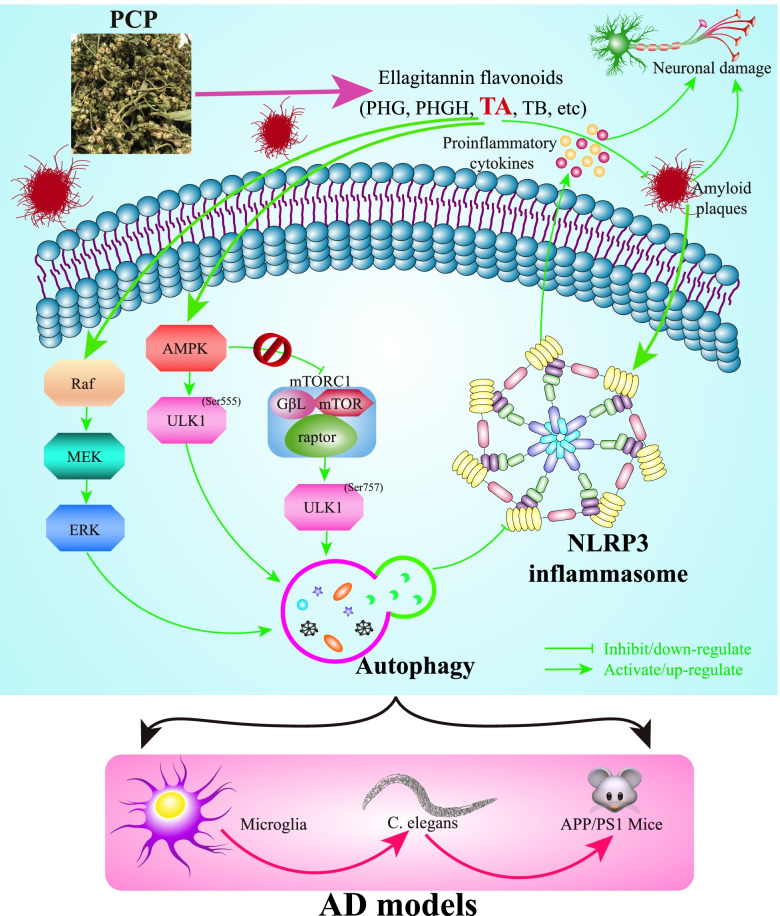
The schematic diagram depicts that TA, one of the ellagitannin flavonoids derived from PCP, inhibits the activation of the NLRP3 inflammasome via the AMPK/ULK1- and Raf/MEK/ERK-mediated autophagy in the in vitro and in vivo models of AD, including microglia, *C. elegans*, and APP/PS1 mice

In conclusion, this present study revealed that TA derived from PCP could promote the autophagic degradation of the NLRP3 inflammasome in Aβ(1–42)-induced BV-2 cells via the AMPK/ULK1 and Raf/MEK/ERK signaling pathways and improve the cognitive and behavioral functions in *C. elegans* and APP/PS1 mice (Fig. 9), which may provide a potential novel therapeutic reagent for TA in the prevention and treatment of AD in the future.

## Supplementary Information

**Additional file 1.**

## Data Availability

The MS data obtained in this work are available from the corresponding authors on reasonable request.
