# Supplementary material for: Targeting microglial autophagic degradation of the NLRP3 inflammasome for identification of thonningianin A in Alzheimer’s disease
Source: Inflamm Regen. 2022 Aug 3;42:25. doi: 10.1186/s41232-022-00209-7 (PMC9347127; doi:10.1186/s41232-022-00209-7)
Supplement: Supplementary file 1 — Additional file 1. [file 41232_2022_209_MOESM1_ESM.docx]

**Additional file 1.**

**Targeting microglial autophagic degradation of the NLRP3 inflammasome for identification of thonningianin A in Alzheimer’s disease**

Xiao-Gang Zhou^1†^, Wen-Qiao Qiu^1,2†^, Lu Yu^1,†^, Rong Pan^1^, Jin-Feng Teng^1^, Zhi-Pei Sang^3^, Betty Yuen-Kwan Law^4^, Ya Zhao^1^, Li Zhang^1^, Lu Yan^1^, Yong Tang^1,4^, Xiao-Lei Sun^5^, Vincent Kam Wai Wong^4^, Chong-Lin Yu^1^, Jian-Ming Wu^1*^, Da-Lian Qin^1*^ and An-Guo Wu^1*^

*^1^ Sichuan Key Medical Laboratory of New Drug Discovery and Drugability Evaluation, Luzhou Key Laboratory of Activity Screening and Druggability Evaluation for Chinese Materia Medica, Key Laboratory of Medical Electrophysiology of Ministry of Education, School of Pharmacy, Southwest Medical University, Luzhou, 646000, China*

*^2^ Department of Neurosurgery Sichuan Provincial People’s Hospital, University of Electronic Science and Technology of China, Chengdu, 610000, China*

*^3^ School of Pharmaceutical Sciences, Hainan University, Haikou, 570228, China*

*^4^* *State Key Laboratory of Quality Research in Chinese Medicine; Macau University of Science and Technology, Macau, China*

*^5^* *Vascular Surgery Department, Affiliated Hospital of Southwest Medical University, Luzhou, 646000, China*

**^†^**These authors contributed equally to this work.

*****Correspondence to: Jian-Ming Wu; jianmingwu@swmu.edu.cn, Da-Lian Qin; Email: dalianqin@swmu.edu.cn and An-Guo Wu; Email: wuanguo@swmu.edu.cn, Tel: 17769617417.

**Additional File 1.**

**Additional Materials and Methods**

**Additional Figures 1-31**

**Additional Tables 1-6**

**Additional Video 1**

**Additional Materials and Methods**

**Chemicals, plasmids, and antibodies**

LPS was purchased from solarbio Bioscience & Technology Co., Ltd. (Shanghai, China). TAK-242 was obtained from Sigma-Aldrich (Shanghai, China). The antibody against TLR4 (14358) was purchased from Cell Signaling Technologies Inc. (CST, Beverly, MA, USA). ELISA assay kits, including IL-1β (RX203063M), IL-18 (RX203064M), Aβ (RX202395M), TREM2 (RX203266M), NLRP3 (RX202453M), and GFAP (RX202088M) were purchased from Quanzhou Ruixin Biological Technology Co., Ltd. (Quanzhou, China).

**In vitro phagocytosis assay**

BV-2 cells were plated into 6 well plates at a density of 50,000 cells per well and incubated at 37 °C. with 5% CO2. After 24 h, cells were pretreated with TA under the indicated concentrations for 12 h before the addition of carboxylated latex beads (FluoSpheres, 1 μm; Aladdin, Cat#M122073) in a 1:100 cell:bead ratio for an additional 12 h. After treatment, the medium was replaced with fresh medium, BV2 cells were then subjected to examination and captured under a Nikon ECLIPSE 80i fluorescence microscope. The phagocytosis index was calculated as a percentage of the number of cells that had phagocytosed 1 or more latex beads divided by the total number of cells per field.

**ELISA**

After treatment, the cell-free supernatants or brain tissue were collected and subsequently employed for the measurement of IL-1β, Aβ, NRLP3, IL-18, TREM2, and GFAP expression using enzyme-linked immunosorbent assay (ELISA) kits according to the manufacturer’s instructions.

**Compound match and target finding**

Download the molecular structure information of TA in the Pubchem database, use ChemBio3D Ultra 14.0 software to draw the three-dimensional chemical structure of TA, and perform energy minimization, and finally save it as a mol2 format file. Using pharmacophores to match target servers, PharmMapper virtually screens the potential targets of TA ([1](#_ENREF_1)), the target range is set to "Human Protein Targets Only", according to the matching score, the reverse molecular docking score Fit score is the standard, and the first 72 targets are selected as the research object.

**Construction of PPIN and screening of key targets**

To clarify the synergy of potential targets, the STRING platform was used to construct a protein interaction network (PPIN) ([2](#_ENREF_2)), the protein source was set to "Homo sapiens", the lowest interaction threshold was set to "Medium confidence", and the remaining parameters were kept default. The protein interaction network was imported into Cytoscape 3.5.1 software to analyze the network topology attributes. Using degree and betweenness as the screening conditions ([3](#_ENREF_3)), nodes with degree values and betweenness values above the network average degree value and average betweenness values are the key targets, and their functions are studied.

**Analysis of key target functions**

Analyze TA key targets in the KEGG database (Kyoto Encyclopedia of Genes and Genomes) and GO database (Gene Ontology), construct a target pathway network diagram, and analyze the path relationship involved in TA potential targets.

**Molecular docking verification**

Use molecular docking software SYBLY to molecularly dock TA with key targets. SYBLY’s molecular docking program has the characteristics of high accuracy and fast speed. Generally, the docking score <4.25 indicates that the affinity of the molecule and the target is not strong, and the docking score> 4.25 indicates the molecule has a moderate affinity with the target, a docking score>5 indicates that the molecule has a strong affinity with the target, and a docking score>7 indicates that the molecule has a much stronger affinity with the target. This study uses the docking score to verify the affinity of TA with key targets.

**Thioflavin-T (ThT) Fluorescence Assay**

The direct inhibition of TA on Aβ fibril formation can be evaluated by determining the level of fluorescence intensity by Thioflavin-T (ThT) assay as previously described ([4](#_ENREF_4)). In brief, 20 µL of Aβ(1-42) (100 µM) was mixed with 80 µL of PBS with or without TA and incubated at 37 ℃ in the dark. At the time points of 24, 48, and 72 h, 10 µL per sample were mixed with 190 µL of ThT solution (20 µM) and added into the wells of a black 96-well plate, which was followed by incubation in the dark at 37 ℃ for 1 h. After that, the fluorescence intensity indicating Aβ fibrillization was measured by a Cytation 3 microplate reader at an excitation wavelength of 450 nm and an emission wavelength at 490 nm. Meanwhile, the background fluorescence of the control solution containing PBS and 0.02% DMSO was subtracted.

**Biolayer Interferometry (BLI) Analysis**

In this study, we used the BLI analysis to detect the direct binding of Aβ with TA as previously descried ([4](#_ENREF_4)). In brief, 200 µL of Aβ(1-42) solution (50 µM) was firstly prepared. Then, EZ-Link NHS-LC-LC-Biotin (Thermo Scientific, United States) was prepared in DMSO with the final concentration being 10 mM. Aβ and biotin reagents were biotinylated in a 3:1 molar ratio of biotin reagent and incubated at room temperature for 30 min. Then, the biotinylated Aβ(1-42) solution, which was filtered by the Zeba desalting spin column, was added into the wells of a 96-well plate (Greiner BioOne, PN:656206). Biotinylation was ascertained by loading the mixture onto streptavidin (SA) capacity tips (ForteìBIO, Menlo Park, CA, USA) and detected by the ForteìBIO, Octet Red 96e instrument (ForteìBIO, Menlo Park, CA, USA) according to the manufacturer’s instructions. In addition, all SA biosensors were pre-wetted with PBS containing 5% DMSO and 0.02% Tween for 10 min. The SA sensor with or without fixed Aβ is equilibrated in PBS containing 5% DMSO and 0.02% Tween. Then, 200 µL of TA with the concentrations of 6.25, 12.5, 25, 50, 100, and 200 µM were added into the wells. Meanwhile, the solution containing an equal amount of DMSO as the control was also added into the wells. All experiments were composed of repeated cycles of four major steps: baseline (120 s), loading (180 s), association (100 s), and dissociation (100 s). The association and dissociation curves and kinetic constants were analyzed and calculated by ForteìBIO data analysis software (ForteìBIO, Menlo Park, CA, USA).

**Fig. S1**


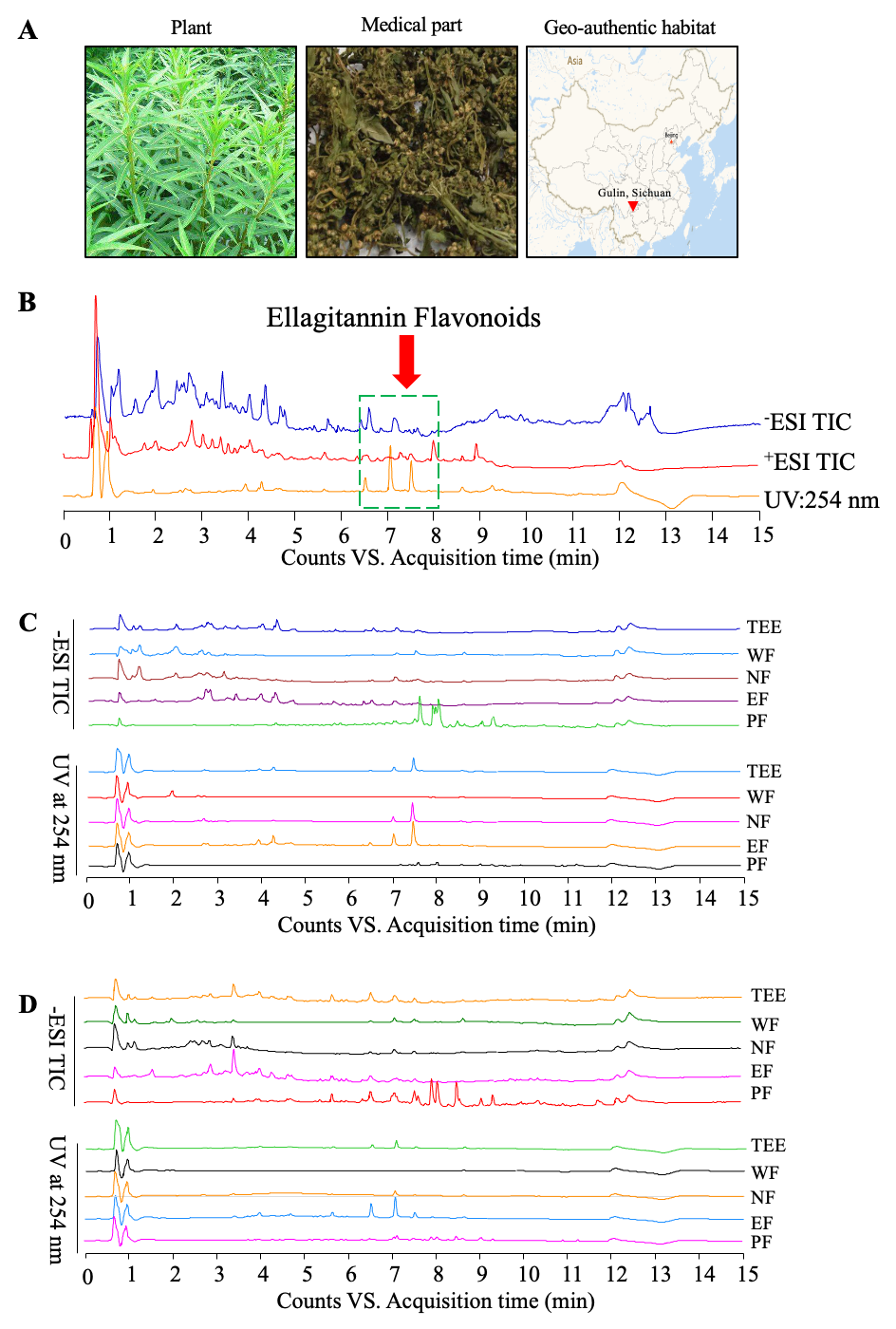


**Fig. S1** Identification of the components in the PCP-TEE and its derived fractions. **A** The aerial botanical morphology and dried medical material of PCP, and its genuine origin is in Gulin, Sichuan province of China. **B** Chromatograms of PCP flower TEE were obtained by UHPLC-DAD-TOF/MS in negative and positive ion modes and monitored by a UV detector at 254 nm, respectively. **C** Total ion chromatogram (TIC) in negative ion mode and DAD chromatogram at 254 nm of PCP leaf TEE and its extracted water fraction (WF), n-Butanol fraction (NF), ethylethanoate fraction (EF), and petroleum ether fraction (PF). **D** TIC in negative ion mode and DAD chromatogram at 254 nm of PCP stem TEE and its extracted WF, NF, EF, and PF.

**Fig. S2**


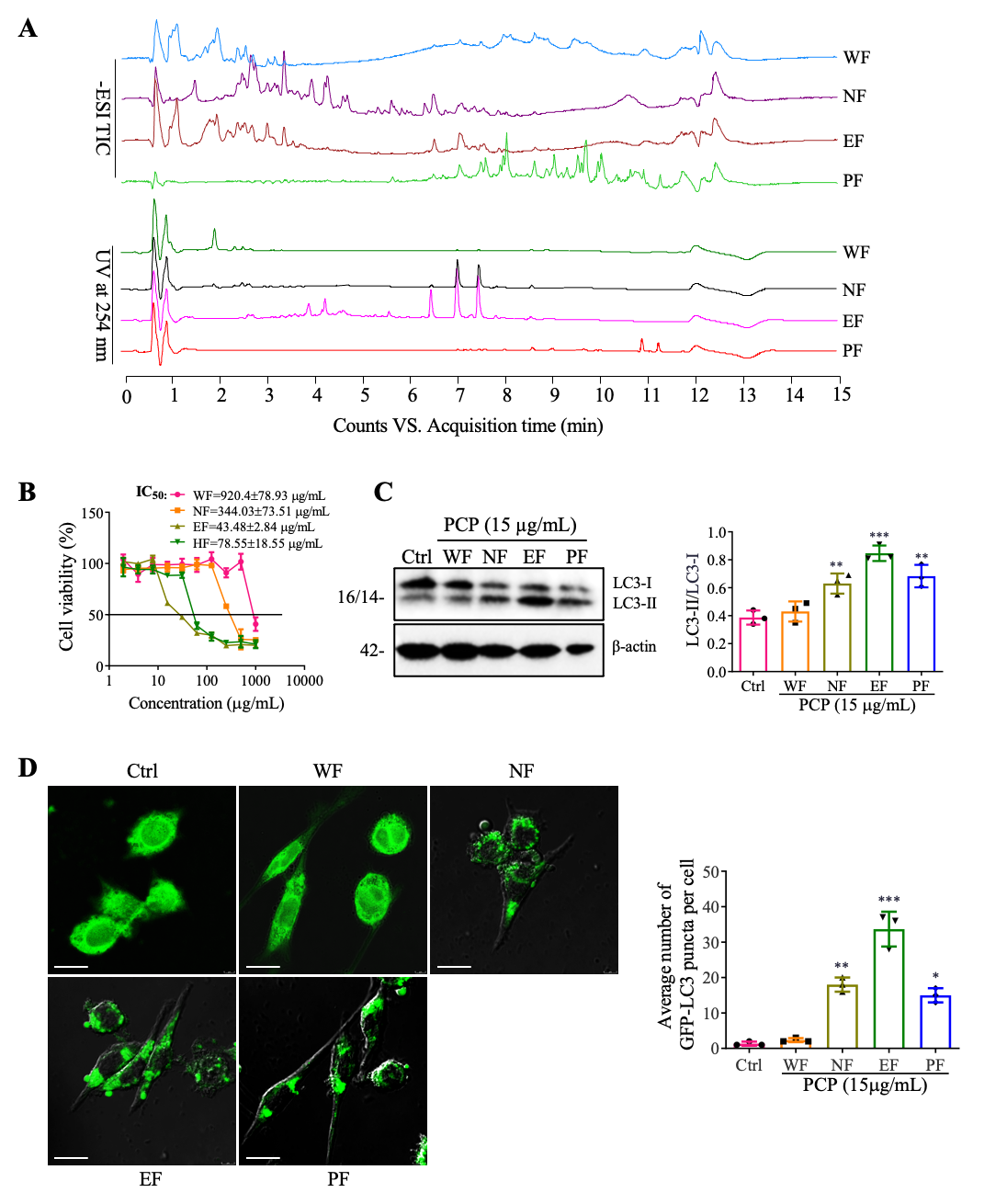


**Fig. S2** EF of PCP-TEE induces autophagy in BV-2 cells. **A** Representative TIC in negative ion mode and DAD chromatograms at 254 nm of WF, NF, EF, and PF from PCP flower TEE. **B** The cytotoxicity of WF, NF, EF, and PF from PCP flower TEE in BV-2 cells was measured by MTT assay, and their IC_50_ values were calculated using the Prism 5.0 software (*n*=3). **C** Protein expression of LC3 in BV-2 cells treated with 15 µg/mL of WF, NF, EF, and PF from PCP flower TEE for 24 h. The bar chart indicates the ratio of LC3-II/LC3-I (*n*=3). The full-length blots are presented in Fig. S27. **D** Representative fluorescence images of BV-2 cells transfected with GFP-LC3 plasmid and treated with 15 µg/mL of WF, NF, EF, and PF from PCP flower TEE were captured. Magnification, ×63; Scale bar: 25 µm. The bar chart represents the average number of GFP-LC3 puncta per cell (*n*=3). Error bars, S.D. **p* ≤ 0.05; ***p* ≤ 0.01; ****p* ≤ 0.001. (One-way ANOVA with Tukey-corrected post-hoc t-test for multiple comparisons was applied for comparison between groups).

**Fig. S3**


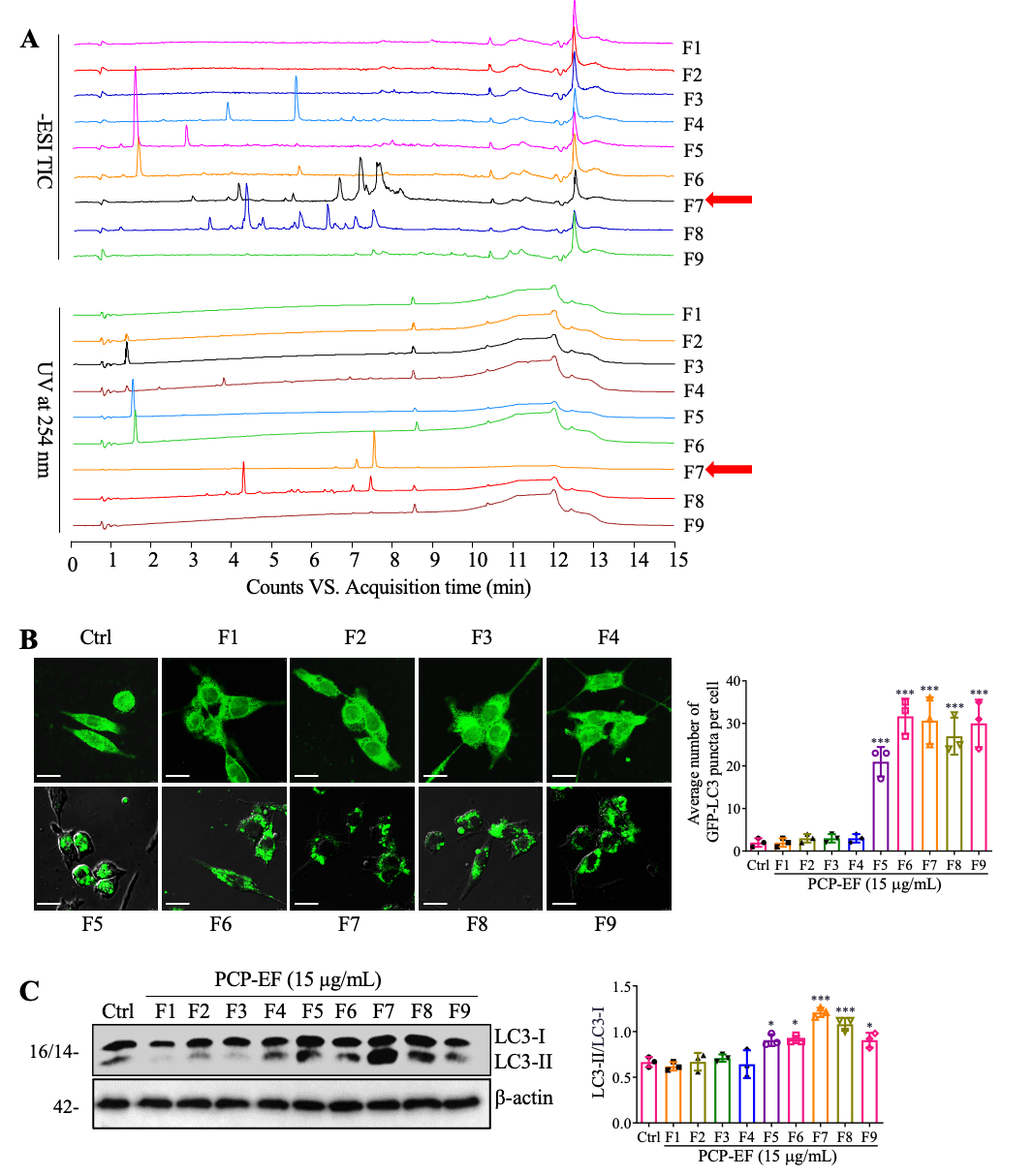


**Fig. S3** The UHPLC-DAD-TOF-MS analysis and autophagy induction effect of the sub-fractions isolated from the EF of PCP-TEE. **A** TIC in ion negative mode and DAD chromatograms at 254 nm of F1-F9 isolated from the EF of PCP-TEE. **B** Representative fluorescence images of BV-2 cells transfected with GFP-LC3 plasmid and treated with 15 µg/mL F1-F9 for 24 h were captured. The bar chart indicates the average number of GFP-LC3 puncta per cell (*n*=3). Magnification, ×63; Scale bar: 25 µm. **C** Protein expression of LC3 in BV-2 cells treated with 15 µg/mL F1–F9 for 24 h. The bar chart indicates the ratio of LC3-II/LC3-I (*n*=3). Error bars, S.D. **p* ≤ 0.05, ****p* ≤ 0.001. (One-way ANOVA with Tukey-corrected post-hoc t-test for multiple comparisons was applied for comparison between groups). The full-length blots are presented in Fig. S27.

**Fig. S4**


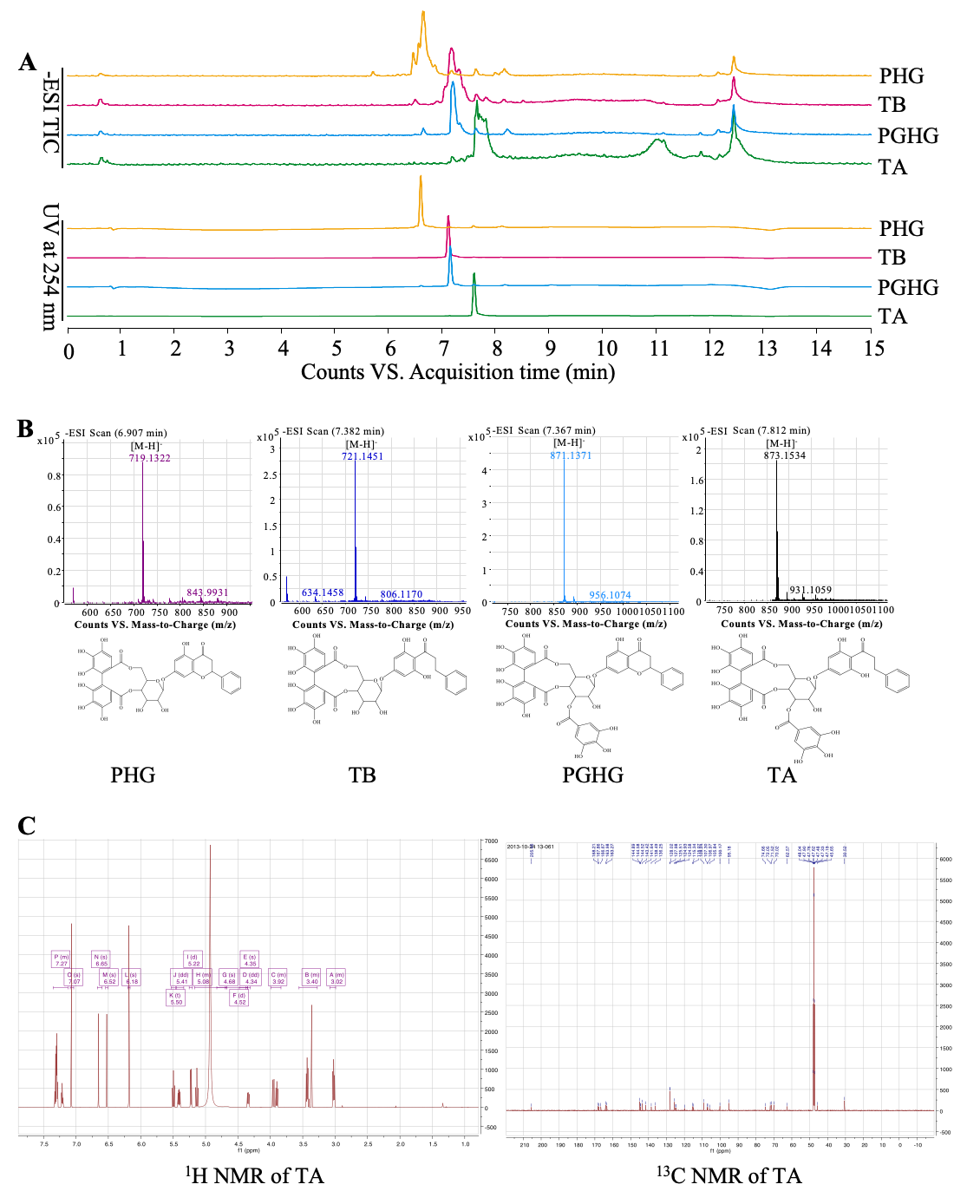


**Fig. S4** Isolation and identification of PHG, TB, PGHG, and TA. **A** Representative TIC in negative ion mode and DAD chromatograms at 254 nm of PHG, TB, PGHG, and TA. **B** The mass spectrums of PHG, TB, PGHG, and TA in negative ion mode and their molecular structures. **C** ^1^H NMR and ^13^C NMR spectrums of TA.

**Fig. S5**

**Fig. S5** PHG, TB, PGHG, and TA induce autophagy in BV-2 cells. **A** Cytotoxicity of PHG, TB, PGHG, and TA in BV-2 cells was measured by MTT assay, and their IC_50_ values were calculated using the Prism 5.0 software (*n*=3). **B** Protein expression of LC3 in BV-2 cells treated with 10 µM of PHG, TB, PGHG, and TA for 24 h. The bar chart indicates the ratio of LC3-II/LC3-I (*n*=3). The full-length blots are presented in Fig. S27. **C** Representative fluorescence images of BV-2 cells transfected with GFP-LC3 plasmid and treated with 10 µM of PHG, TB, PGHG, and TA for 24 h were captured. The bar chart represents the average number of GFP-LC3 puncta per cell (*n*=3). Magnification, ×63; Scale bar: 25 µm. Error bars, S.D. **p* ≤ 0.05, ***p* ≤ 0.01, ****p* ≤ 0.001. (One-way ANOVA with Tukey-corrected post-hoc t-test for multiple comparisons was applied for comparison between groups).

**Fig. S6**

**Fig. S6** TA induces autophagy in BV-2 and MEF cells. **A** Representative fluorescence images of BV-2 cells transfected with GFP-LC3 plasmid and treated with 10 µM of TA for 0, 6, 12, and 24 h were captured. The bar chart represents the average number of GFP-LC3 puncta per cell (*n*=4). Magnification, ×63; Scale bar: 25 µm. **B** Representative images of BV-2 cells transfected with tfLC3 plasmid and treated with 2.5, 5, and 10 µM of TA or 0.5 µM of Rap for 24 h were captured. The bar chart indicates the GFP/RFP fluorescence ratio of BV-2 cells (*n*=3). Magnification, ×63; Scale bar: 25 µm. **C** The cytotoxicity of TA in Atg7^+/+^ and Atg7^-/-^ MEF cells was measured by MTT assay (*n*=3). **D** Protein expression of LC3 in Atg7^+/+^ and Atg7^-/-^ MEF cells treated with 2.5, 5, and 10 µM of TA for 24 h. The bar chart indicates the ratio of LC3-II/LC3-I (*n*=3). Error bars, S.D. ***p* ≤ 0.01; ****p* ≤ 0.001. (One-way ANOVA with Tukey-corrected post-hoc t-test for multiple comparisons was applied for comparison between groups). The full-length blots are presented in Fig. S28.

**Fig. S7**

**Fig. S7** CC, SBI, or SCH inhibits TA-induced LC3-II/LC3-I conversion. **A**, **B**, **C** BV-2 cells were pretreated with CC (10 µM), SBI (40 µM), or SCH (20 µM) for 1 h, followed by the treatment of TA (10 µM) for an additional 24 h. Then the protein expression of LC3 in BV-2 cells was detected using Western blot. Bar charts indicate the ratio of LC3-II/LC3-I (*n*=3). Error bars, S.D. **p* ≤ 0.05; ****p* ≤ 0.001. (One-way ANOVA with Tukey-corrected post-hoc t-test for multiple comparisons was applied for comparison between groups). The full-length blots are presented in Fig. S29.

**Fig. S8**

**Fig. S8** AMPK/ULK1 and Raf/MEK/ERK signaling pathways interact and synergistically contribute to the regulation of TA-induced autophagy in BV-2 cells. **A**, **B** BV-2 cells were pretreated with CC (10 µM) or SCH (20 µM) for 1 h, followed by the treatment of TA (10 µM) for an additional 24 h. Then protein expressions were detected using Western blot. Bar charts indicate the ratios of p-AMPK/AMPK, p-ULK1(555)/ULK1, and p-ERK/ERK (*n*=3). **C** Protein expression of BV-2 cells pretreated with SCH (40 µM), CC (10 µM), or SCH (40 µM) with CC (10 µM) for 1 h and followed by treatment with TA (10 µM) for an additional 24 h. The bar chart indicates the ratio of LC3-II/LC3-I (*n*=3). Error bars, S.D. **p* ≤ 0.05; ***p* ≤ 0.01; ****p* ≤ 0.001. (One-way ANOVA with Tukey-corrected post-hoc t-test for multiple comparisons was applied for comparison between groups). The full-length blots are presented in Fig. S30.

**Fig. S9**

**Fig. S9** The effect of TA on the morphology and phagocytic capacity of BV-2 cells. **A** Representative images of BV-2 cells treated with TA (2.5, 5, and 10 µM) for 24 h. **B** Representative merged white light and fluorescence images of BV-2 cells pretreated with TA under the indicated concentrations for 12 h before the addition of carboxylated latex beads for an additional 12 h. The bar chart indicates the phagocytosis index of BV-2 cells. Error bars, S.D. (One-way ANOVA with Tukey-corrected post-hoc t-test for multiple comparisons was applied for comparison between groups).

**Fig. S10**

**Fig. S10** Quantification of band intensity of Western Blot in Fig. 3A (**A**) and Fig. 3B (**B**). Bar charts indicate the relative protein expression of NLRP3/β-actin, ASC/β-actin, caspase-1 (p10)/pro-caspase-1, IL-1β (p17)/pro-IL-1β, IL-18 (p18)/pro-IL-18, and GSDMD (p30)/pro-GSDMD in BV-2 cells (n=3). Error bars, S.D. *p ≤ 0.05; **p ≤ 0.01; ***p ≤ 0.001. (One-way ANOVA with Tukey-corrected post-hoc t-test for multiple comparisons was applied for comparison between groups).

**Fig. S11**

**Fig. S11** ELISA assay of IL-1β in BV-2 cells. BV-2 cells were pretreated with 5 µM of Aβ(1-42) for 12 h, followed by treatment with 2.5, 5, and 10 µM of TA or inhibitors including LY, CC, and SCH for an additional 24 h. Cell supernatants were then collected for the measurement of released IL-1β using an ELISA kit according to the manufacturer’s instructions. The bar chart indicates the level of IL-1β (*n*=6). Error bars, S.D. **p* ≤ 0.05; ***p* ≤ 0.01; ****p* ≤ 0.001. (One-way ANOVA with Tukey-corrected post-hoc t-test for multiple comparisons was applied for comparison between groups).

**Fig. S12**

**Fig. S12** TA inhibits the level of IL-1β and NLRP3 in mouse primary microglial cells. Mouse primary microglial cells were pretreated with 5 µM of Aβ(1-42) for 12 h, followed by the treatment of TA at the indicated concentration for an additional 24 h. Cell supernatants were then collected for the measurement of (**A**) IL-1β and (**B**) NLRP3 using ELISA kits according to the manufacturer’s instructions. Bar charts indicate the level of IL-1β and NLRP3 (*n*=3). Error bars, S.D. **p* ≤ 0.05; ***p* ≤ 0.01; ****p* ≤ 0.001. (One-way ANOVA with Tukey-corrected post-hoc t-test for multiple comparisons was applied for comparison between groups).

**Fig. S13**

**Fig. S13** The effect of TA, CC, LY, SCH, TA + CC, TA + LY, or TA + SCH on the cell viability of PC-12 cells. PC-12 cells were treated with TA (10 µM), CC (10 µM), LY (10 µM), SCH (20 µM), TA (10 µM) + CC (10 µM), TA (10 µM) + LY (10 µM), or TA (10 µM) + SCH (20 µM) for 24 h. Then the cell viability of PC-12 was measured by MTT assay. The bar chart indicates the cell viability of PC-12 cells. Error bars, S.D. (One-way ANOVA with Tukey-corrected post-hoc t-test for multiple comparisons was applied for comparison between groups).

**Fig. S14**

**Fig. S14** TA inhibits Aβ fibrillization and its induced neuronal damage and induces autophagy in mouse primary hippocampal neurons. **A** The bar chart indicates the ThT fluorescence of Aβ(1-42) (20 µM) solution alone or mixed solutions containing Aβ(1-42) and TA (*n*=7). **B** Kinetic measurement of the binding affinity of TA to Aβ by BLI assay. Kinetic binding sensor grams at increasing concentrations of TA from 6.25 to 200 μM. The response (nm) represents the optical thickness of the sensor layer, which is reflected by the spectral shift (Δλ) under the interaction of TA with Aβ(1-42). The response at steady state shows that the rate of association was equal to that of dissociation. The equilibrium binding signal (Req) indicated by a flattened curve was reached. The kinetic constants, including the values of KD, Kon, and Kids, calculated by the ForteìBIO data analysis software are shown in the table. **C** Representative Hoechst 33342/PI staining images of mouse primary hippocampal neurons treated with TA (10 µM) in the absence or presence of 5 µM of Aβ(1-42) for 24 h. Magnification, ×20; Scale bar: 100 µm. The bar chart indicates the ratio of PI/Hoechst signals of cells (*n*=3). **D** Representative fluorescence images of mouse primary hippocampal neuron cells transfected with GFP-LC3 plasmid and treated with TA and Rap at the indicated concentrations for 24 h. Magnification, ×63; Scale bar: 25 µm. The bar chart represents the average number of GFP-LC3 puncta per cell (*n*=3). Error bars, S.D. **p* ≤ 0.05; ****p* ≤ 0.001. (One-way ANOVA with Tukey-corrected post-hoc t-test for multiple comparisons was applied for comparison between groups).

**Fig. S15**

**Fig. S15.** ELISA assay of Aβ, IL-1β, NLRP3, IL-18, TERM2, and GFAP in the brain tissue of NC and APP/PS1 mice. Bar charts indicate the level of Aβ, IL-1β, NLRP3, IL-18, TERM2, and GFAP (*n*=4). Error bars, S.D. **p* ≤ 0.05; ***p* ≤ 0.01; ****p* ≤ 0.001. (One-way ANOVA with Tukey-corrected post-hoc t-test for multiple comparisons was applied for comparison between groups).

**Fig. S16**


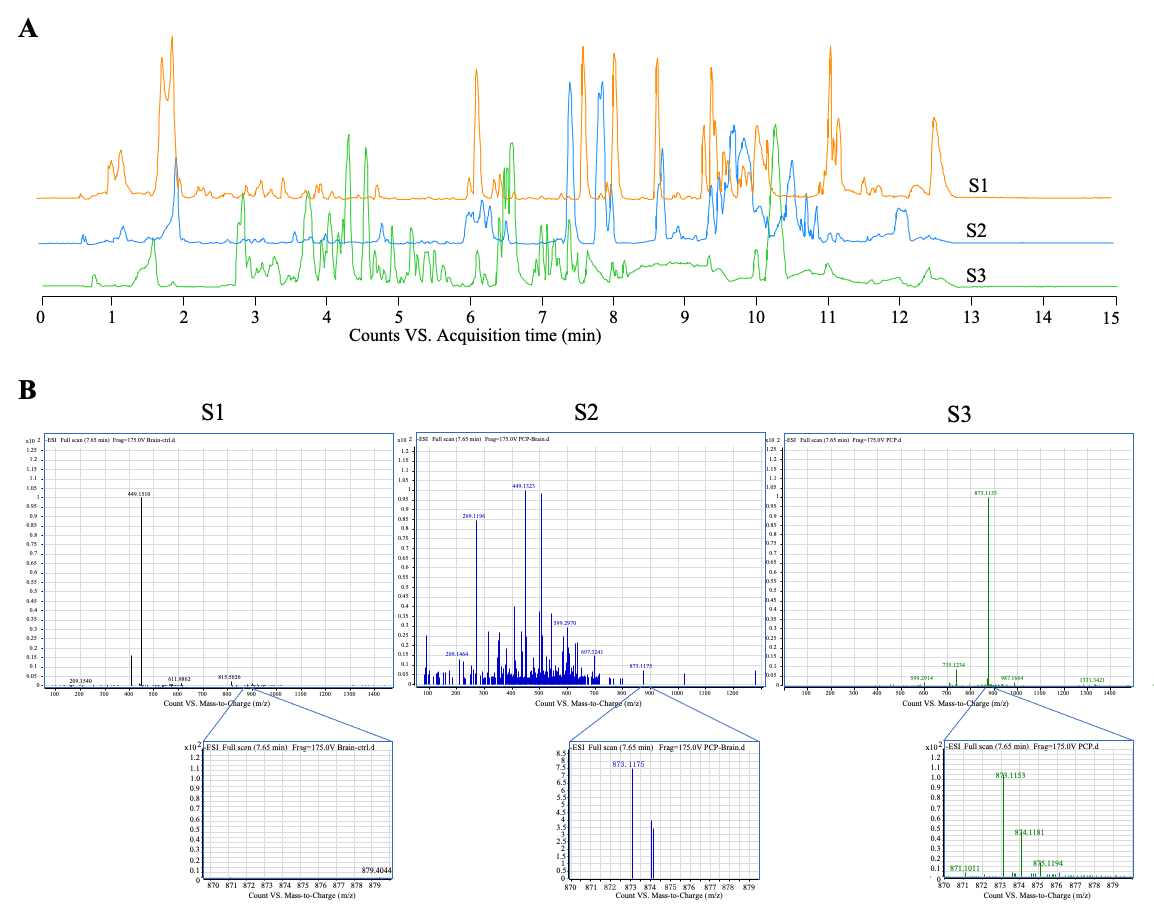


**Fig. S16** UHPLC-DAD-TOF/MS analysis of TA in the brain tissue. **A** TIC of S1, S2, and S3. **B** The mass spectrum of TA in S1, S2, and S3, respectively. S1: brain of vehicle mice; S2: brain of mice intraperitoneally administrated with PCP-TEE; S3: PCP-TEE.

**Fig. S17**

**Fig. S17** The target predication of TA by network pharmacology theory. **A** Using the Pharmacophore PharmMapper matching target server, SwissTargetPrediction and SEA virtual to screen 72 potential targets of TA, with the auxiliary of the STRING platform construction of target-protein interaction network. **B**, **C** Using KEGG and GO analysis to explore the function of the target database and the pathways, the relationship between diseases. **D** SYBYL molecular docking system server TA and key targets for molecular docking, high score target protein with STRING platform construction target protein with AMPK/ULK1 and RAF/MEK/ERK pathway protein interaction networks. **E** Molecular docking of HSP90AA1 with TA.

**Fig. S18**

**Fig. S18** TA inhibited NLRP3 inflammasome independent of TLR4. BV-2 cells were treated with 10 µM of LPS for 12 h, followed by the treatment of TA (10 µM) or TAK (10 µM) for an additional 24 h. Then protein expressions were detected using the Western blot. Bar chart indicates the ratios of TLR4/β-actin, NLRP3/β-actin, ASC/β-actin, caspase-1 (p10)/Pro, IL-1β (p17)/Pro, IL-18 (p18)/ β-actin, and GSDMD (p30)/Pro (*n*=3). Error bars, S.D. **p* ≤ 0.05; ***p* ≤ 0.01; ****p* ≤ 0.001. (One-way ANOVA with Tukey-corrected post-hoc t-test for multiple comparisons was applied for comparison between groups). The full-length blots are presented in Fig. S31.

**Fig. S19**

**Fig. S19** The full-length Western blotting images of Fig. 1A, B, E, F.

**Fig. S20**

**Fig. S20** The full-length Western blotting images of Fig. 2A, B.

**Fig. S21**

**Fig. S21** The full-length Western blotting images of Fig. 3A.

**Fig. S22**

**Fig. S22** The full-length Western blotting images of Fig. 3B.

**Fig. S23**

**Fig. S23** The full-length Western blotting images of Fig. 4A, B.

**Fig. S24**

**Fig. S24** Full-images of DA2123 worms treated with vehicle, TA, or Rap as indicated concentration in Fig. 6C.

**Fig. S25**

**Fig. S25** The full-length Western blotting images of Fig. 7B.

**Fig. S26**

**Fig. S26** The full-length Western blotting images of Fig. 8A, C.

**Fig. S27**

**Fig. S27** The full-length Western blotting images of Fig. S2C, 3C, 5B.

**Fig. S28**

**Fig. S28** The full-length Western blotting images of Fig. S6D.

**Fig. S29**

**Fig. S29** The full-length Western blotting images of Fig. S7.

**Fig. S30**

**Fig. S30** The full-length Western blotting images of Fig. S8.

**Fig. S31**

**Fig. S31** The full-length Western blotting images of Fig. S18.

**Table S1.** Identification of the compounds in PCP flower part by UHPLC-DAD-TOF/MS.

| **Peak No.** | **RT (min)** | **Negative mode** | **Accurate mass** | **Positive mode** | **Accurate mass** | **Proposed compounds** |
| --- | --- | --- | --- | --- | --- | --- |
| 1 | 1.066 | [M-H]^-^ | 355.0337 | [M+H]^+^ | 357.0785 | Chebulic acid |
| 2 | 2.803 | [M-H]^-^ | 289.077 | [M+H]^+^ | 291.0861 | Epicatechin |
| 3 | 2.895 | [M-H]^-^ | 291.0816 | [M+H]^+^ | 293.0841 | Brevifolin-carboxylic acid |
| 4 | 3.194 | [M-H]^-^ | 329.0938 | [M+H]^+^ | 331.1142 | 2,6-dihybroxyacetophenone-4-o-β-D-glucoside |
| 5 | 3.344 | [M-H]^-^ | 625.1471 | [M+H]^+^ | 627.1531 | Quercetin di-O-glycoside |
| 6 | 3.661 | [M-H]^-^ | 607.0961 | [M+H]^+^ | 609.1152 | Penthorumin C |
| 7 | 3.678 | [M-H]^-^ | 609.1481 | [M+H]^+^ | 611.1596 | Rutin |
| 8 | 3.827 | [M-H]^-^ | 301.0047 | [M+H]^+^ | 303.0421 | Quercetin |
| 9 | 4.052 | [M-H]^-^ | 593.1149 | [M+H]^+^ | 595.1594 | Kaempferol-3-O-rutinosde |
| 10 | 4.071 | [M-H]^-^ | 431.1044 | [M+H]^+^ | 433.1492 | 5-methoxy-pinocembrin-7-O-glucoside |
| 11 | 4.151 | [M-H]^-^ | 431.1044 | [M+H]^+^ | 433.1492 | Kaempferol-3-O-rhamnoside |
| 12 | 4.152 | [M-H]^-^ | 433.085 | [M+H]^+^ | 435.0926 | Quercetin-3-O-xyloside |
| 13 | 4.244 | [M-H]^-^ | 433.0856 | [M+H]^+^ | 435.1134 | Quercetin-3-O-arabinopyranoside |
| 14 | 4.236 | [M-H]^-^ | 447.1062 | [M+H]^+^ | 449.1072 | Quercetin-3-O-rhamnoside isomer |
| 15 | 4.311 | [M-H]^-^ | 447.1087 | [M+H]^+^ | 449.1078 | Quercetin-3‘-O-rhamnoside isomer |
| 16 | 4.652 | [M-H]^-^ | 603.1089 | [M+H]^+^ | 605.1125 | Unidentified |
| 17 | 4.669 | [M-H]^-^ | 631.0999 | [M+H]^+^ | 633.1088 | 2,6-dihydroxyacetophenone-4-O-[4',6'-(S)-hexahydroxydiphenoyl]-β-D-glucose |
| 18 | 4.735 | [M-H]^-^ | 603.1089 | [M+H]^+^ | 605.1125 | Unidentified |
| 19 | 5.26 | [M-H]^-^ | 619.0992 | [M+H]^+^ | 621.1234 | Unidentified |
| 20 | 5.352 | [M-H]^-^ | 619.0999 | [M+H]^+^ | 621.1254 | Unidentified |
| 21 | 5.666 | [M-H]^-^ | 417.125 | [M+H]^+^ | 419.2643 | Pinocembrin-7-glucoside |
| 22 | 5.702 | [M-H]^-^ | 419.1418 | [M+H]^+^ | 421.1136 | Unidentified |
| 23 | 5.907 | [M-H]^-^ | 569.1359 | [M-H]^-^ | 571.1421 | Pinocembrin-7-O-galloyl-D-glucoside |
| 24 | 6.043 | [M-H]^-^ | 885.1519 | [M+H]^+^ | 887.1345 | Unidentified |
| 25 | 6.351 | [M-H]^-^ | 419.1418 | [M+H]^+^ | 421.1136 | Unidentified |
| 26 | 6.509 | [M-H]^-^ | 719.1379 | [M+H]^+^ | 721.1162 | Pinocembrin-7-O-[4'',6''-HHDP]-glucoside |
| 27 | 6.589 | [M-H]^-^ | 269.0853 | [M+H]^+^ | 271.1551 | Apigenin |
| 28 | 7.055 | [M-H]^-^ | 721.1404 | [M+H]^+^ | 723.1235 | Pinocembrin dihydrochalcone-7-O-[4'',6''-HHDP]-glucoside |
| 29 | 7.059 | [M-H]^-^ | 871.1467 | [M+H]^+^ | 873.1254 | Pinocembrin-7-O-[3''-O-galloyl-4'',6''-HHDP]-glucoside |
| 30 | 7.542 | [M-H]^-^ | 873.1573 | [M+H]^+^ | 875.1536 | Pinocembrin dihydrochalcone-7-O-[3''-O-galloyl-4'',6''-HHDP]-glucoside |
| 31 | 7.862 | [M-H]^-^ | 885.162 | [M+H]^+^ | 887.1243 | Unidentified |
| 32 | 7.967 | [M-H]^-^ | 861.1615 | [M+H]^+^ | 863.0239 | Unidentified |
| 33 | 8.017 | [M-H]^-^ | 887.16 | [M+H]^+^ | 889.0331 | Unidentified |
| 34 | 8.198 | [M-H]^-^ | 255.0962 | [M+H]^+^ | 257.0807 | Pinocembrin |
| 35 | 8.608 | [M-H]^-^ | 463.0324 | [M+H]^+^ | 464.0864 | Quercetin-3-O-β-D-glucoside |

Note: RT, retention time

**Table S2.** The relative percent (%) of PHG, TB, PGHG, and TA in PCP flower, leaf, and stem.

| **Compounds**  **FCP-parts** | **PHG (%)** | **TB (%)** | **PGHG (%)** | **TA (%)** |
| --- | --- | --- | --- | --- |
| Flower | 56.41 | 51.63 | 64.77 | 39.48 |
| Leaf | 13.37 | 39.79 | 17.61 | 55.87 |
| Stem | 30.22 | 8.58 | 17.61 | 4.65 |
| Total | 100 | 100 | 100 | 100 |

**Table S3.** The relative percent (%) of PHG, TB, PGHG and TA in the fractions isolated from PCP flower.

| **Compounds**  **Fractions** | **PHG (%)** | **TB (%)** | **PGHG (%)** | **TA (%)** |
| --- | --- | --- | --- | --- |
| WF | 1.36 | 2.51 | 1.42 | 1.93 |
| NF | 5.36 | 14.44 | 28.91 | 30 |
| EF | 90.92 | 78.91 | 67.53 | 64.35 |
| PF | 2.35 | 4.15 | 2.14 | 3.72 |
| Total | 100 | 100 | 100 | 100 |

**Table S4.** The relative percent (%) of PHG, TB, PGHG, and TA in the fractions isolated from PCP leaf.

| **Compounds**  **Leaf-fractions** | **PHG (%)** | **TB (%)** | **PGHG (%)** | **TA (%)** |
| --- | --- | --- | --- | --- |
| WF | 4.52 | 2.66 | 3.98 | 2.95 |
| NF | 8.76 | 13.89 | 29.55 | 35.82 |
| EF | 81.91 | 79.43 | 62.30 | 56.61 |
| PF | 4.82 | 4.02 | 4.17 | 4.62 |
| Total | 100 | 100 | 100 | 100 |

**Table S5.** The relative percent (%) of PHG, TB, PGHG, and TA in the fractions isolated from PCP stem.

| **Compounds**  **Leaf-fractions** | **PHG (%)** | **TB (%)** | **PGHG (%)** | **TA (%)** |
| --- | --- | --- | --- | --- |
| WF | 1.07 | 5.17 | 1.89 | 2.12 |
| NF | 3.64 | 9.40 | 10.25 | 13.64 |
| EF | 92.59 | 78.15 | 83.93 | 77.12 |
| PF | 2.70 | 7.28 | 3.93 | 7.12 |
| Total | 100 | 100 | 100 | 100 |

**Table S6.** The relative percent (%) of quercetin, PHG, TB, PGHG, and TA in F1-F9

| **Compounds**  **Fractions** | **PHG (%)** | **TB (%)** | **PGHG (%)** | **TA (%)** |
| --- | --- | --- | --- | --- |
| F1 | 0 | 0 | 0 | 0 |
| F2 | 0 | 0 | 0 | 0 |
| F3 | 0 | 0 | 0 | 0 |
| F4 | 83.04 | 0 | 0 | 0 |
| F5 | 12.08 | 0 | 0 | 0.031 |
| F6 | 4.88 | 0.248 | 0.69 | 0.40 |
| F7 | 0 | 74.05 | 48.95 | 86.33 |
| F8 | 0 | 23.72 | 46.31 | 12.26 |
| F9 | 0 | 2.00 | 4.05 | 0.98 |

**Video S1**

**
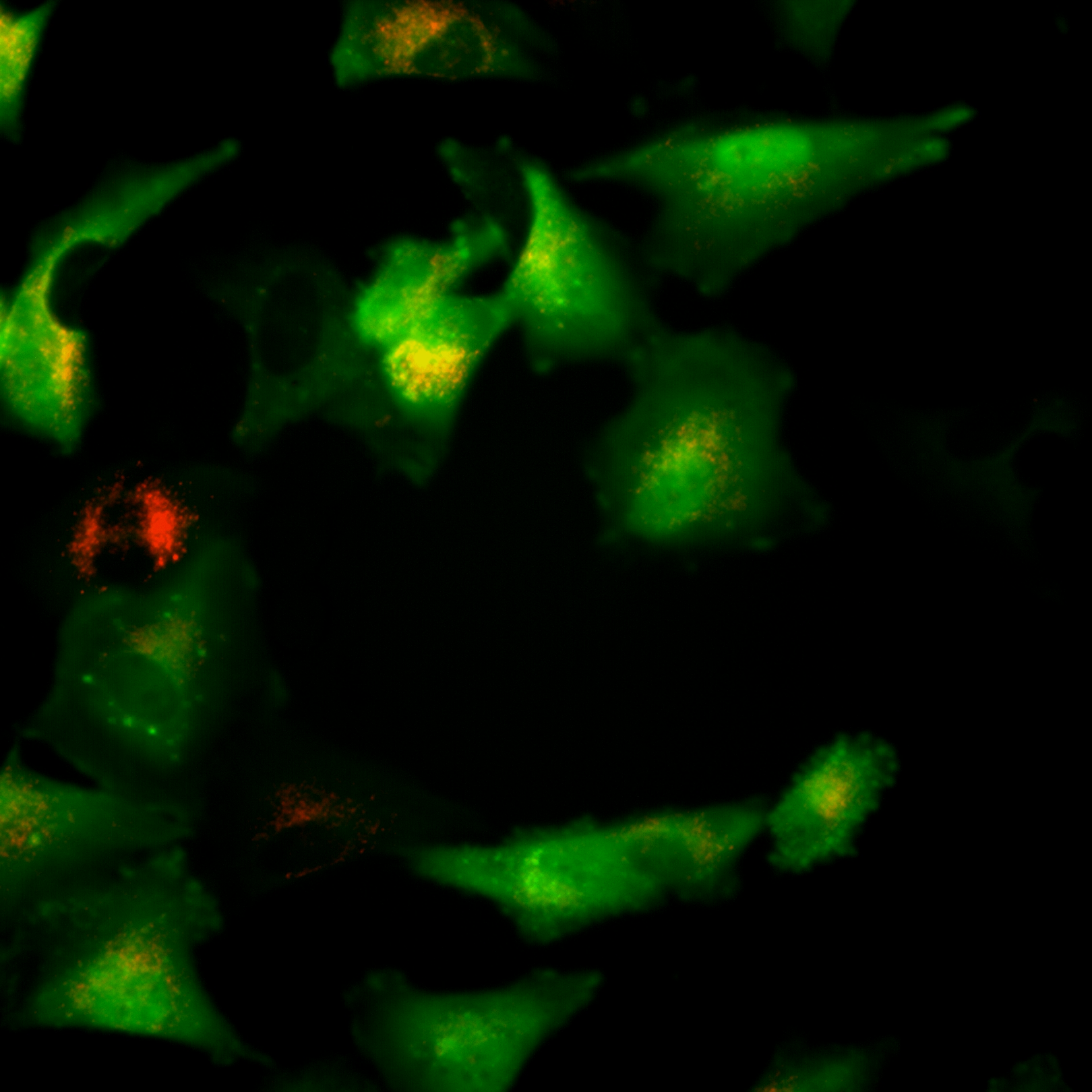
**

**Video S1** The real-time TA-induced autophagy flux in stable RFP-GFP-LC3 U87 cells in 24 h. The dynamic change in RFP-LC3 and GFP-LC3 of stable RFP-GFP-LC3 U87 cells was performed by real-time observation using ImageXpress Micro 4 Widefield High-Content Imaging System (Molecular Devices, San Jose, CA, USA).

**References**

1. Liu X, Ouyang S, Yu B, Liu Y, Huang K, Gong J, et al. PharmMapper server: a web server for potential drug target identification using pharmacophore mapping approach. Nucleic Acids Res. 2010;38(Web Server issue):W609-14.

2. von Mering C, Jensen LJ, Snel B, Hooper SD, Krupp M, Foglierini M, et al. STRING: known and predicted protein-protein associations, integrated and transferred across organisms. Nucleic Acids Res. 2005;33(Database issue):D433-7.

3. Li YH, Yu CY, Li XX, Zhang P, Tang J, Yang Q, et al. Therapeutic target database update 2018: enriched resource for facilitating bench-to-clinic research of targeted therapeutics. Nucleic Acids Res. 2018;46(D1):D1121-D7.

4. Yu L, Wu AG, Wong VK, Qu LQ, Zhang N, Qin DL, et al. The New Application of UHPLC-DAD-TOF/MS in Identification of Inhibitors on beta-Amyloid Fibrillation From Scutellaria baicalensis. Front Pharmacol. 2019;10:194.
